# A Chitosan-Based Neuropeptide Hydrogel with Zinc Oxide-Assisted Antioxidant Activity for Ultraviolet B-Induced Delayed Wound Repair

**DOI:** 10.34133/bmr.0443

**Published:** 2026-11-09

**Authors:** Hanying Wang, Qian Chen, Guizhu Feng, Qiqi Pei, Jingpei Shi, Yu Zhang, Chengjie Ma, Yubin Hu, Shiwei Niu, Lixian Mu, Lechun Lyu

**Affiliations:** ^1^ Yunnan Key Laboratory of Plateau Thermal Medical Rehabilitation and Wellness, School of Rehabilitation, Kunming Medical University, Kunming 650500, Yunnan, China.; ^2^ School of Basic Medical Sciences, Kunming Medical University, Kunming 650500, Yunnan, China.; ^3^ School of Nursing, Kunming Medical University, Kunming 650500, Yunnan, China.; ^4^ Department of Orthopedics, The First People’s Hospital of Yunnan Province and the Affiliated Hospital of Kunming University of Science and Technology, Kunming 650500, Yunnan, China.; ^5^ Department of Medical Imaging, First Affiliated Hospital of Kunming Medical University, Kunming 650500, Yunnan, China.; ^6^ Yunnan Key Laboratory of Stem Cell and Regenerative Medicine, College of Rehabilitation, Kunming Medical University, Kunming 650500, Yunnan, China.

## Abstract

Chronic and delayed-healing wounds are often sustained by persistent inflammation and oxidative stress, which impair cell migration, angiogenesis, and tissue remodeling. In this study, we developed a chitosan (CS)-based neuropeptide hydrogel that combines local delivery of substance P (SP) with ZnO-assisted antioxidant activity. SP was grafted onto CS, and the self-assembling peptide Nap-β was introduced to form a fibrillar hydrogel network for local retention of SP-related components. ZnO nanoparticles were incorporated to reinforce the hydrogel structure and improve antioxidant protection. The resulting CS-SP-Nap-β@ZnO hydrogel showed appropriate gelation behavior, an interconnected porous morphology, self-healing properties, sustained release behavior, and acceptable cytocompatibility and hemocompatibility. In vitro, the hydrogel reduced inflammatory cytokine production, promoted macrophage transition toward a reparative phenotype, attenuated reactive oxygen species accumulation and oxidative injury, and supported the proliferation and migration of wound-related cells. In an ultraviolet B-induced delayed-healing wound model, CS-SP-Nap-β@ZnO accelerated wound closure and promoted re-epithelialization, collagen deposition, and neovascularization. These findings suggest that CS-SP-Nap-β@ZnO can improve the wound repair microenvironment by combining SP-mediated inflammatory regulation with ZnO-assisted oxidative stress control.

## Introduction

Chronic skin wounds are becoming increasingly common as chronic metabolic diseases and population aging continue to rise, creating a substantial burden for patients, clinicians, and healthcare systems [1,2]. Current treatments include ultrasonic hydro-debridement, negative pressure wound therapy, platelet-rich plasma, and local delivery of cytokines, stem cells, or pharmacological agents; however, the clinical response is often incomplete and difficult to sustain [3]. Unlike acute wounds, chronic wounds frequently remain trapped in a self-perpetuating inflammatory phase. Excessive inflammatory mediators and reactive oxygen species (ROS) accumulation impair the migration of wound-related cells, inhibit angiogenesis, and delay extracellular matrix remodeling [4–8]. In addition to metabolic and infectious factors, ultraviolet B (UVB)-induced photodamage can further intensify this process by generating additional ROS, amplifying inflammatory signaling, and weakening the activity of keratinocytes, fibroblasts, endothelial cells, and immune cells [9]. A local microenvironment characterized by both inflammatory persistence and oxidative stress therefore represents a central obstacle to delayed wound repair. Accordingly, strategies that coordinate inflammation control with redox regulation may offer a more effective route for improving chronic or delayed wound healing than approaches that address either process alone.

Against this background, we sought a bioactive factor that could participate in the wound-healing cascade while also helping to rebalance the inflammatory microenvironment. Database screening focused our attention on substance P (SP), a neuropeptide encoded by the tachykinin precursor 1 (TAC1) gene that has well-established roles in neuroimmune communication and tissue repair. SP acts mainly through the G protein-coupled neurokinin-1 receptor (NK-1R) and has been reported to influence immune-cell recruitment, macrophage phenotype, keratinocyte migration, endothelial activation, and angiogenesis [10–15]. These biological effects make SP an attractive candidate for restoring a more reparative wound microenvironment. Nevertheless, the direct use of soluble SP is limited by rapid proteolytic degradation and poor retention at the wound surface, making it difficult to maintain an effective local concentration over time [16,17]. A delivery platform that can immobilize SP at the wound site and release it gradually is therefore needed to translate its biological activity into a practical dressing system.

Oxidative stress provides another major barrier to effective wound repair. When ROS production exceeds local antioxidant defenses, cell proliferation is suppressed, inflammatory signaling is reinforced, and tissue regeneration is delayed [18]. Although endogenous antioxidant enzymes can remove ROS, their limited stability, short functional lifetime, and potential immunogenicity restrict their direct therapeutic use [19]. Synthetic nanozymes with enzyme-like antioxidant activity have therefore attracted considerable interest in wound-healing applications [11,20–22]. Among these materials, metal-organic frameworks (MOFs) are appealing because their metal coordination sites can provide catalytic activity and drug-loading capacity [23,24]. MOF-based nanozymes have shown promise in diabetic wound repair and angiogenesis promotion [25]. However, conventional MOF systems may still raise concerns related to structural rigidity, uncontrolled metal ion release, and ligand-associated biosafety [26–29]. For local wound care, a softer hydrogel-based coordination environment may better balance catalytic function, hydration, degradability, and tissue compatibility [30].

To meet these requirements, we designed a chitosan (CS)-based neuropeptide hydrogel that integrates sustained SP delivery with ZnO-assisted antioxidant activity for chronic wound repair (Fig. 1). CS was chosen as the polymeric backbone because it is biocompatible, biodegradable, and chemically amenable to peptide conjugation [31–35]. SP was grafted onto the CS chain to improve its local retention, while the self-assembling peptide motif NapFFVLEGG (Nap-β) was introduced to generate a fibrillar network [36]. This architecture was intended to hold SP within the hydrogel matrix and release it in a sustained manner. Zinc oxide nanoparticles were incorporated into the CS-peptide network to reinforce the hydrogel structure and provide ZnO-assisted antioxidant activity [37–41]. This design allowed the hydrogel to serve as both a local dressing and a carrier for inflammatory and oxidative stress regulation. The resulting CS-SP-Nap-β@ZnO hydrogel was expected to support wound repair through 2 complementary mechanisms: SP-mediated regulation of inflammatory responses and ZnO-assisted control of oxidative stress. In vitro, the hydrogel alleviated oxidative injury, promoted macrophage transition toward a reparative phenotype, and enhanced the proliferation and migration of wound-related cells. In a UVB-induced delayed-healing wound model, CS-SP-Nap-β@ZnO accelerated wound closure and improved re-epithelialization, collagen deposition, and neovascularization. Together, these findings support a multifunctional dressing strategy in which sustained neuropeptide delivery and antioxidant microenvironment regulation are combined to improve chronic wound repair.

**Fig. 1.**
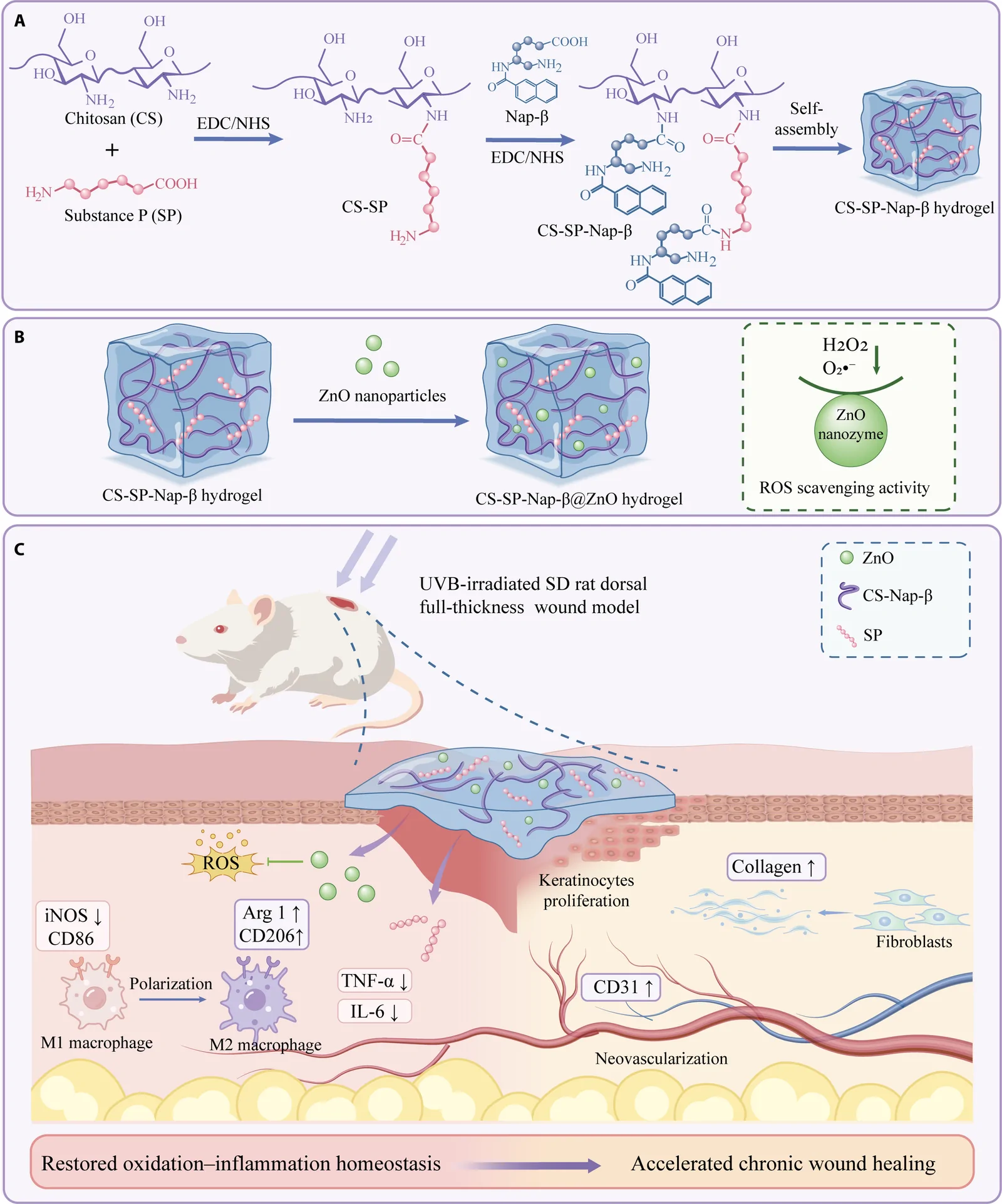
Preparation and application of CS-SP-Nap-β@ZnO self-assembled hydrogel. (A) Synthesis of CS-SP-Nap-β@ZnO hydrogel. (B) Hydrogel wound treatment schematic diagram. (C) Application of CS-SP-Nap-β@ZnO hydrogel in UVB-preconditioned full-thickness wounds.

## Materials and Methods

### Materials

SP (RPKPQQFFGLM) and Nap-β (NapFFVLEGG) were synthesized by DanGang Bioscience (China). ZnO nanoparticles (50 ± 10 nm) were purchased from Solarbio Bioscience (China), and CS (>90% deacetylation; approximately 200 kDa) from Macklin (China). N-hydroxysuccinimide (NHS), 1-ethyl-3-(3-dimethylaminopropyl) carbodiimide hydrochloride (EDC), glacial acetic acid, and dimethyl sulfoxide (DMSO) were purchased from Aladdin (China). Calcein-AM/propidium iodide (PI), lipopolysaccharide (LPS), interferon-γ (IFN-γ), interleukin-4 (IL-4), and other routine cell-culture reagents were obtained from Beyotime. CCK-8 and enzyme-linked immunosorbent assay (ELISA) kits were purchased from Neobioscience, and the EdU Cell Proliferation Kit was obtained from Sigma-Aldrich. Aprepitant was purchased from MedChemExpress. Antibodies against inducible nitric oxide synthase (iNOS) and Arg1 were purchased from Proteintech. Antibodies against NK-1R, AKT, p-AKT, ERK1/2, and p-ERK1/2 were purchased from HUABIO. The Zn Colorimetric Assay Kit was purchased from Elabscience.

RAW264.7 cells were laboratory-maintained; human umbilical vein endothelial cells (HUVECs), human immortalized epidermal cells (HaCaT) cells, and human skin fibroblasts (HSFs) were obtained from the Chinese Academy of Sciences. Culture reagents were purchased from Gibco. Male Sprague–Dawley rats (180 to 220 g; 5 to 6 weeks) were supplied by Kunming Medical University and housed under specific pathogen-free (SPF) conditions at 20 to 22 °C. Animal procedures followed the *Guide for the Care and Use of Laboratory Animals* and were approved by the Animal Ethics Review Committee of Kunming Medical University (kmmu20230114).

### Molecular docking of SP with NK-1R

The experimentally determined structure of NK-1R was retrieved from the RCSB Protein Data Bank (PDB ID: 7P02; cryo-electron microscopy structure at a resolution of 2.87 A). Prior to docking, the original SP ligand, Gs protein, and other nonreceptor components were excluded, and only the NK-1R structure was retained. Mature SP has the amino acid sequence RPKPQQFFGLM, with a naturally amidated C terminus. The one-letter amino acid sequence of SP was submitted to HPEPDOCK for global blind protein–peptide docking using default parameters. HPEPDOCK accounted for the conformational flexibility of SP by generating multiple peptide conformations and ranked the resulting complex models according to the HPEPDOCK docking score. The highest-ranked model was selected as the representative predicted binding pose and then submitted to PRODIGY to estimate the binding free energy. PyMOL version 3.0.1 was used to analyze and visualize the predicted binding interface, interacting residues, and hydrogen bonds.

### Biological activity of SP

SP-induced HUVEC migration was evaluated by scratch assay. Confluent HUVECs in 6-well plates were cultured in medium containing 1% fetal bovine serum (FBS) for 12 h. After scratching and phosphate-buffered saline (PBS) washing, cells were incubated in serum-free medium with SP (10 μg/ml), vascular endothelial growth factor (VEGF, 50 ng/ml, positive control), or aprepitant (5 μM). Images were captured at identical positions at 0 and 24 h, and wound closure was quantified using ImageJ.

For anti-inflammatory analysis, mouse peritoneal macrophages and RAW264.7 cells were stimulated with LPS (100 ng/ml) and treated with SP (5, 10, or 20 μg/ml). After 24 h, supernatant tumor necrosis factor-α (TNF-α), IL-6, and IL-1β levels were measured by ELISA. Cellular proteins were extracted using RIPA buffer, quantified by bicinchoninic acid (BCA) assay, separated by sodium dodecyl sulfate–polyacrylamide gel electrophoresis, and transferred to nitrocellulose membranes. Membranes were blocked with 5% skim milk for 1 h, incubated overnight at 4 °C with anti-iNOS (1:1,000) and anti-Arg1 (1:5,000), followed by horseradish peroxidase-conjugated secondary antibodies, and visualized by chemiluminescence.

For viability analysis, HSFs were seeded in 96-well plates and treated with SP (0, 2, 5, 10, or 20 μg/ml) for 24 h. Cell viability was determined by CCK-8 assay at 450 nm. Subsequent ELISA and Western blotting analyses examining the mechanism underlying the action of SP were performed as described above, with minor procedural modifications. SP, aprepitant, and LPS were used at final concentrations of 10 μg/ml, 5 μM, and 200 ng/ml, respectively. For Western blotting, primary antibodies were diluted as follows: NK-1R (1:5,000), AKT (1:4,000), p-AKT (1:4,000) ERK1/2 (1:4,000), p-ERK1/2 (1:4,000), and GAPDH (1:50,000).

SP was mixed 1:1 with mouse plasma (final concentration, 10 μg/ml) and incubated at 37 °C for 0 to 180 min. At each time point, proteins were precipitated with methanol and the supernatant was collected. Residual SP bioactivity was assessed by its ability to support HSF viability using the CCK-8 assay.

### Synthesis and characterization of CS-SP-Nap-β@ZnO

CS-SP-Nap-β was synthesized by EDC/NHS-mediated amidation. CS (500 mg) was dissolved in 50 ml of ice-cold 1% acetic acid and stirred overnight. SP (500 μg) in 5 ml of deionized water was activated with EDC (150 μg) and NHS (60 μg), added to the CS solution, and reacted overnight at 4 °C. Nap-β (5 mg) in DMSO was similarly activated, added to the CS-SP mixture, and reacted for 8 h at 4 °C. The product was dialyzed (molecular weight cutoff [MWCO], 3,500 Da) for 72 h and lyophilized.

For hydrogel formation, lyophilized CS-SP-Nap-β (0.25 g) was dissolved in 8 ml of deionized water, stirred overnight, and incubated at 37 °C. Gelation was confirmed by tube inversion. CS-SP-Nap-β@ZnO was prepared by adding ZnO (5 μg/ml) to the precursor, stirring for 30 min, and lyophilizing. Material structure and morphology were characterized by Fourier transform infrared spectroscopy (FT-IR), ultraviolet–visible spectroscopy (UV–Vis), proton nuclear magnetic resonance spectroscopy (¹H NMR), scanning electron microscopy (SEM), and transmission electron microscopy (TEM).

### Determination of grafting efficiency and degree of substitution

SP and Nap-β were quantified by UV–Vis spectroscopy using their characteristic absorbance at 258 and 278 nm, respectively. Calibration curves were generated using SP and Nap-β solutions of known concentrations. After the coupling reaction, unreacted SP and Nap-β in the collected dialysates were determined using the respective calibration curves. Grafting efficiency and degree of substitution were calculated as follows:

$$\text{Grafting efficiency}\;\left(\%\right)=m_{0}-m_{f}/m_{0}\times 100$$ (1)

$$\begin{array}{c} \text{degree of substitution}\;\left(\mathrm{DS}\right)\;\left(\mathrm{mol}\%\right)=\\ \left(m_{0}-m_{f}\right)/M_{s}/m_{\mathrm{CS}}/M_{r}\times 100 \end{array}$$ (2)

where *m*_0_ is the initial mass of SP or Nap-β, *m*_f_ is the corresponding unreacted mass detected in the dialysate, *M*_s_ is the molecular weight of SP or Nap-β, *m*_CS_ is the mass of CS, and *M*_r_ is the molecular weight of the CS glucosamine repeat unit.

### Evaluation of hydrogel physical properties

Self-healing, injectability, and adhesion were assessed by reconnecting 2 cut hydrogel pieces, 1 stained with methylene blue, extruding the hydrogel from a 5-ml syringe, and attaching it to different substrates, respectively. Mechanical properties were measured using a universal testing machine. For tensile testing, specimens with an initial gauge length of 10 mm and a cross sectional area of 8 mm^2^ were stretched at 10 mm/min until failure. For compression testing, cylindrical specimens measuring 10 mm in diameter and 4 mm in height were compressed at 1 mm/min to approximately 90% strain.

Hydrogel rheology was analyzed at room temperature using a Haake Mars60 rheometer. CS-SP-Nap-β gelation was monitored by time sweep. Amplitude sweeps were performed on CS-SP-Nap-β (50 and 100 μg/ml) and CS-SP-Nap-β@ZnO (5 and 10 μg/ml). After formulation screening, CS-SP-Nap-β (100 μg/ml) and CS-SP-Nap-β@ZnO (5 μg/ml) were evaluated by frequency sweep and self-healing tests.

Swelling, water retention, and degradation were evaluated gravimetrically at 37 °C. Freeze-dried samples were weighed (W0), immersed in PBS, and collected at predetermined times. After removing surface water, the swollen weight (Wt) was recorded to calculate the swelling ratio. Fully swollen hydrogels were maintained at 37 °C and weighed at scheduled times to determine water loss. For degradation, hydrogels were shaken in 8 ml of PBS at 37 °C, lyophilized at each time point, and weighed to calculate the remaining mass.

### Release of SP and ZnO from the hydrogels

SP release from the CS-SP-Nap-β and CS-SP-Nap-β@ZnO hydrogels was quantified by UV–Vis spectroscopy at 258 nm using a calibration curve generated from SP solutions ranging from 5 to 500 μg/ml. To account for potential interference from Nap-β, an SP-free CS-Nap-β hydrogel was subjected to the same release procedure and served as the matrix blank. The absorbance of blank release medium at 258 nm was subtracted from that of the corresponding SP-containing sample at each time point. Hydrogel samples (20 mg) were placed in dialysis bags with an MWCO of 3,500 Da and immersed in 3 ml of PBS (pH 5.5) at 37 °C with agitation at 250 rpm. At predefined time points, aliquots of the release medium were collected and replaced with equal volumes of fresh PBS. Cumulative SP release was calculated from the corrected absorbance values with correction for medium replacement.

For the ZnO release assay, CS-SP-Nap-β@ZnO hydrogels (20 mg) were directly immersed in 3 ml of PBS (pH 5.5) without dialysis bags and incubated under the same conditions. A ZnO calibration curve was established based on the absorbance of ZnO nanoparticles at 372 nm. Release medium was collected at predefined time points and replenished with equal volumes of fresh PBS. Cumulative ZnO release was calculated from the calibration curve with correction for medium replacement. Soluble Zn^2+^ in the release medium was separately quantified using a Zinc (Zn) Colorimetric Assay Kit (Elabscience Biotechnology Co., Ltd., Wuhan, China) according to the manufacturer’s instructions.

### In vitro biocompatibility of the hydrogel

Hydrogel cytocompatibility was evaluated using CCK-8 and Calcein-AM/PI live/dead staining assays. HSFs were seeded in 96-well plates and treated with SP, Nap-β, CS-SP, ZnO nanoparticles, or hydrogel samples at the indicated concentrations for 48 h. Cell viability was then measured using the CCK-8 assay. For live/dead staining, HSFs and HaCaT cells were seeded in 12-well plates and cocultured with different hydrogel samples for 48 h. The cells were stained with Calcein-AM and PI according to the manufacturer’s instructions and observed under a fluorescence microscope.

Blood compatibility was assessed using a hemolysis assay. Rat blood was centrifuged at 4 °C and 3,000 rpm for 15 min to collect red blood cells (RBCs). The RBCs were washed repeatedly with PBS and resuspended to obtain a 2% RBC suspension. For each group, 500 μl of the RBC suspension was mixed with 500 μl of the corresponding sample and incubated at 37 °C for 3 h. Physiological saline and 0.1% Triton X-100 were used as the negative and positive controls, respectively. After centrifugation at 3,500 rpm for 5 min, the absorbance of the supernatant was measured at 540 nm, and the hemolysis rate was calculated.

### In vitro regulation of macrophage phenotype by the hydrogel

Primary mouse peritoneal macrophages were isolated by peritoneal lavage and purified by adherence before use. RAW264.7 cells and primary peritoneal macrophages were used to evaluate the effect of the hydrogel on macrophage phenotype regulation.

For cytoskeletal staining, RAW264.7 cells were seeded at 2 × 10^5^ cells/ml and stimulated with LPS (100 ng/ml) to induce an inflammatory phenotype. The cells were then treated with SP, CS-Nap-β, CS-SP-Nap-β, or CS-SP-Nap-β@ZnO. IL-4 (20 ng/ml) was used as a positive control for M2-like polarization. After 48 h of culture, macrophages were stained with fluorescein isothiocyanate (FITC)-phalloidin and Hoechst to visualize the cytoskeleton and nuclei.

For immunofluorescence staining, macrophages were fixed, permeabilized, blocked, and incubated with primary antibodies against CD206 or Arg1, followed by fluorescent secondary antibodies. Nuclei were counterstained with 4′,6-diamidino-2-phenylindole (DAPI), and images were captured using a fluorescence microscope.

For flow cytometry, primary peritoneal macrophages were treated with the corresponding materials, collected, and stained with antibodies against CD45 (1:400), CD11b (1:400), F4/80 (1:400), CD86 (1:200), CD206 (1:200), and CD163 (1:200). After excluding debris and doublets, CD45^+^CD11b^+^F4/80^+^ cells were gated as macrophages, within which CD86 and CD163/CD206 expression was analyzed to evaluate macrophage polarization. Unstained and single-stained controls were used for background assessment and fluorescence compensation. In parallel, cell lysates or culture supernatants were collected, and TNF-α and IL-6 levels were quantified by ELISA.

### ROS-scavenging activity of ZnO nanoparticles

The O_2_•^−^-scavenging activity of ZnO nanoparticles (1, 2, 5, and 10 μg/ml) was evaluated using a WST-1-based assay according to the manufacturer’s instructions. After incubation with the superoxide-generating reaction system, absorbance was measured at 450 nm, and the O_2_•^−^-scavenging efficiency was calculated relative to the untreated control. Superoxide dismutase (SOD, 2 U/ml) was used as the positive control. To evaluate H_2_O_2_ scavenging, ZnO nanoparticles were incubated with 200 μmol/L H_2_O_2_ at 37 °C for 30 min. After removing the nanoparticles by centrifugation, the residual H_2_O_2_ concentration in the supernatant was determined using a colorimetric H_2_O_2_ assay at 560 nm. Catalase (0.1 U/ml) was used as the positive control.

### In vitro antioxidant activity of the hydrogel

To evaluate antioxidant activity under inflammatory oxidative stress, RAW264.7 cells were treated with the corresponding hydrogels for 48 h, exposed to UVB (1.33 mW/cm^2^, 15 s; 20 mJ/cm^2^), and stimulated with LPS. Controls were untreated, whereas model cells received UVB and LPS without hydrogel protection. Cells were lysed with kit buffer, and protein concentrations were determined by BCA assay. Equal protein amounts were analyzed using a WST-8-based SOD assay at 450 nm. SOD activity was normalized to total protein and expressed as U/mg protein.

For ROS analysis, HaCaT cells were seeded in 6-well plates (5 × 10^5^ cells/well) and assigned to control, H_2_O_2_ model, CS-Nap-β, or CS-SP-Nap-β@ZnO groups. Hydrogel groups were pretreated with the corresponding extracts before exposure to H_2_O_2_ (600 μM, 4 h). Cells were then incubated with the 2′,7′-dichlorodihydrofluorescein diacetate (DCFH-DA) probe at 37 °C for 30 min in the dark and washed with PBS. ROS levels were observed by fluorescence microscopy and quantified by flow cytometry. Apoptosis was evaluated under identical conditions by Annexin V-FITC/PI staining and flow cytometry.

### In vitro wound-healing activity of the hydrogel

Hydrogel effects on HSF and HaCaT proliferation and migration were evaluated using EdU and Transwell assays, respectively. For proliferation, cells were seeded in 12-well plates at 3 × 10^4^ cells/cm^2^ and cultured with UV-sterilized hydrogels for 48 h. EdU-positive cells were labeled with Azide 555, counterstained with Hoechst, imaged, and quantified. For migration, serum-starved cells were seeded in 8-μm Transwell inserts containing serum-free medium. The lower chambers contained 600 μl of complete medium with 20% FBS. After 24 h of hydrogel treatment, migrated cells were fixed, stained with crystal violet, imaged, and quantified in randomly selected fields.

### In vivo wound-healing study

A UVB-preconditioned full-thickness excisional wound model was established in male Sprague–Dawley rats weighing 180 to 220 g. After removal of the dorsal hair, the rats were irradiated using a KN-4006BL2 narrowband UVB phototherapy device (Kernel Medical Equipment Co., Ltd., Xuzhou, China) with a nominal peak wavelength of 311 nm. The irradiance was set to 0.65 mW/cm^2^, and the distance between the UVB source and the dorsal skin was maintained at 15 cm. A shielding template with a 4-cm^2^ exposure window was placed over the dorsum to define the irradiation area. The skin was irradiated for 30 min, corresponding to a cumulative dose of 1.17 J/cm^2^. One week postirradiation, rats were anesthetized with 2.5% pentobarbital sodium (40 mg/kg) and given 1-cm dorsal full-thickness wounds. They were randomized into model, CS-Nap-β, SP, CS-SP-Nap-β, or CS-SP-Nap-β@ZnO groups. Wounds received 200 μl of SP (10 μg/ml) or corresponding hydrogel and were photographed on days 3, 7, 14, and 21. ImageJ quantified wound area relative to baseline. Health was monitored and blood was collected at selected times. Serum pro-inflammatory cytokines and SOD activity were measured using commercial ELISA and SOD assay kits, respectively.

### Histology and immunohistochemistry

Wound tissues were collected at predetermined time points and fixed in 4% paraformaldehyde. Histological analysis included hematoxylin and eosin (H&E) staining and Masson’s trichrome staining performed according to standard protocols. Re-epithelialization and collagen deposition were quantified from H&E- and Masson-stained sections.

Angiogenesis and local immune marker expression in wound tissues were evaluated by immunofluorescence staining for CD31, CD86, and CD206. Tissue sections were blocked and incubated with the corresponding primary antibodies (1:400), followed by fluorescent secondary antibodies (1:500). Nuclei were counterstained with DAPI. Fluorescence images were captured using a fluorescence microscope, and the signals were quantified using ImageJ. To assess preliminary in vivo biosafety, major organs were collected at the end of the experiment, fixed in 4% paraformaldehyde, embedded in paraffin, sectioned, and stained with H&E for histological observation. Serum levels of alanine aminotransferase (ALT) and urea were measured using an automated biochemical analyzer. Serum Zn^2+^ concentrations were measured using a zinc colorimetric assay kit.

### Data analysis

Data are presented as the mean ± SD. ImageJ was used for image quantification. Statistical analysis was performed using one-way analysis of variance (ANOVA) for comparisons among multiple groups. For wound-closure curves measured across different time points, repeated-measures or 2-way ANOVA was used when appropriate. Statistical significance was defined as follows: ns, not significant; *P < 0.05; **P < 0.01; ***P < 0.001; and ****P < 0.0001.

## Results

### Screening of SP and validation of its pro-migratory activity

To screen for secreted factors associated with wound repair, we analyzed differentially expressed mRNAs in skin and oral mucosal wound datasets from the Gene Expression Omnibus (GEO) database. Because oral mucosal wounds usually heal faster than skin wounds, we first compared wound and normal tissues within each tissue type and then performed cross-comparison between skin and oral mucosal wounds. Nine overlapping differentially expressed mRNAs were identified among the 3 comparisons (Fig. 2A and Fig. S1A to C).

**Fig. 2.**
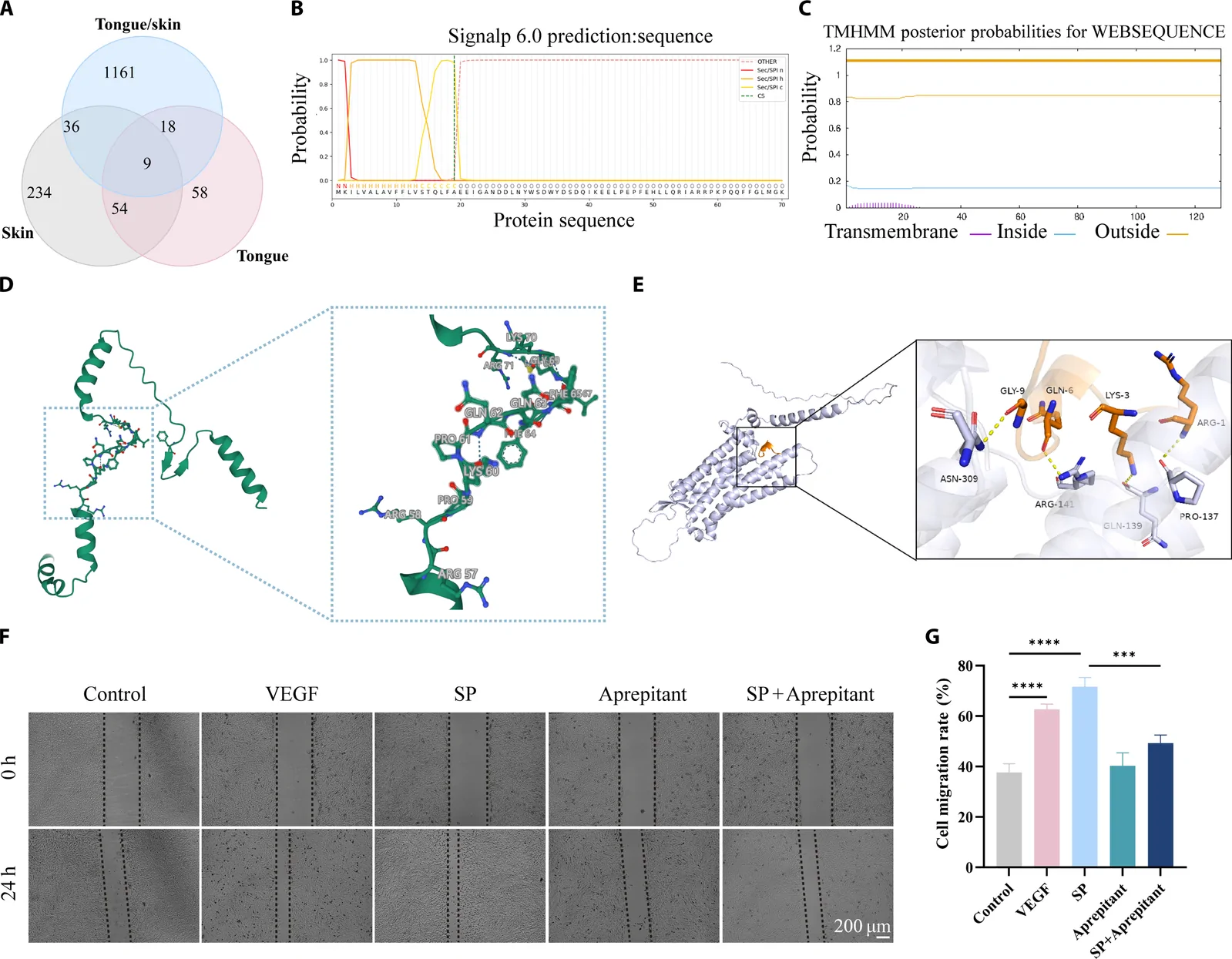
Screening and validation of substance P (SP) as a bioactive peptide associated with wound repair. (A) Overlapping analysis of differentially expressed transcripts from skin and oral mucosal wound datasets. (B) SignalP-based prediction of TAC1. (C) TMHMM-based prediction of TAC1 transmembrane regions. (D) Schematic diagram of SP derived from TAC1 (E) Molecular docking evaluation of SP with NK-1R. (F) Representative images from the HUVEC scratch assay after treatment with SP ,VEGF, or Aprepitant. Scale bar, 200 μm. (G) Quantification of the wound-closure rate. Data are expressed as the mean ± SD (*n* = 3). ns, not statistically significant; *P < 0.05, **P < 0.01, ***P < 0.001, ****P < 0.0001.

We subsequently prioritized candidate factors with predicted secretory features. SignalP and TMHMM assessments indicated that TAC1 carried a signal peptide but did not contain a transmembrane domain, indicating that it may function as a classically secreted factor (Fig. 2B and C). TAC1 encodes several tachykinin peptides, among which residues 58 to 68 correspond to SP (Fig. 2D). Molecular docking and structural visualization further indicated that the predicted SP binding pose was located within the ligand-binding region of NK-1R. PRODIGY estimated a binding free energy of Δ*G* = −10.4 kcal·mol^−1^, suggesting a potentially favorable interaction between SP and NK-1R in the computational model (Fig. 2E). In addition, predicted cleavage analysis suggested that SP-derived fragments may retain potential immunomodulatory and pro-angiogenic activities (Fig. S1D and Tables S1 to S5).

The direct influence of SP on endothelial cellular migration was subsequently assessed. In the HUVEC scratch assay, 10 μg/ml SP significantly enhanced cell migration compared with the control group. This effect was markedly attenuated by aprepitant (Fig. 2F and G). The CCK-8 assay also showed lower cell viability in the SP plus aprepitant group than in the SP group (Fig. S2A). These observations support the role of SP as a bioactive factor associated with wound healing. They also indicate that its effects on HUVEC migration and cell viability are at least partially dependent on NK-1R.

### SP regulates macrophage inflammatory responses and demonstrates modest plasma stability

After verifying the pro-migratory activity of SP, we also assessed whether SP could regulate macrophage-mediated pro-inflammatory responses. RAW264.7 macrophages were stimulated with LPS and treated with different concentrations of SP. LPS stimulation substantially elevated the production of TNF-α, IL-1β, and IL-6, whereas SP treatment lowered these inflammatory cytokines, with 10 μg/ml showing the most evident inhibitory effect (Fig. 3A to C). A similar pattern was observed in primary mouse peritoneal macrophages, where SP also reduced LPS-induced TNF-α, IL-6, and IL-1β release (Fig. 3D to F).

**Fig. 3.**
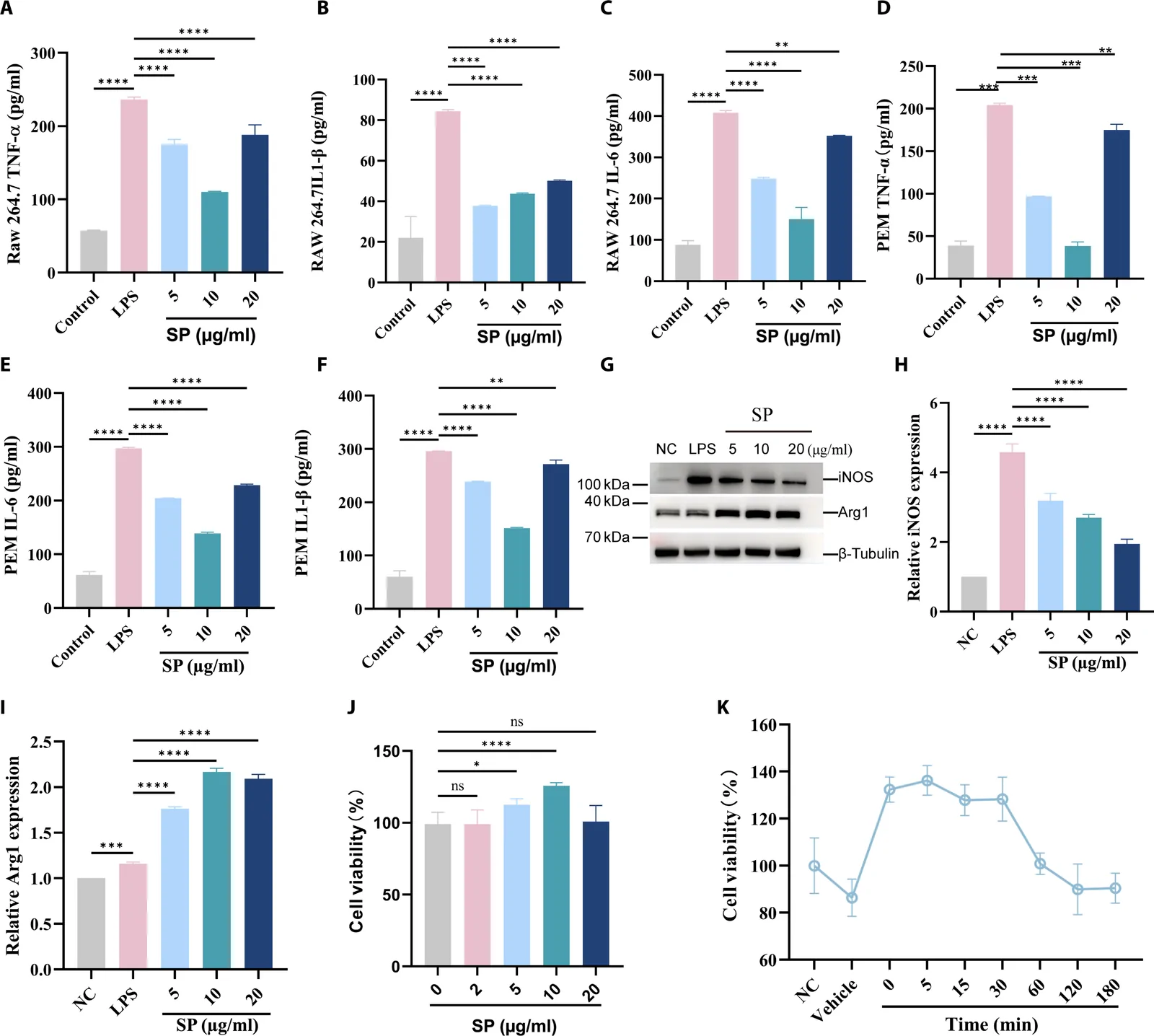
Biological activity and plasma stability of substance P (SP). (A to C) TNF-α, IL-1β, and IL-6 concentrations in LPS-challenged RAW264.7 macrophages following treatment with graded SP concentrations. (D to F) TNF-α, IL-6, and IL-1β concentrations in LPS-challenged primary peritoneal macrophages following SP treatment. (G) Western blotting of iNOS and Arg1 expression. (H and I) Densitometric quantification of iNOS and Arg1 protein levels. (J) CCK-8-based assessment of cell viability following treatment with graded SP concentrations. (K) Cell viability assay following incubation with plasma-exposed SP for different durations. Data are expressed as the mean ± SD (*n* = 3). ns, not statistically significant; *P < 0.05, **P < 0.01, ***P < 0.001, ****P < 0.0001.

Western blot assessment also substantiated the immunomodulatory activity of SP. LPS stimulation up-regulated the M1-associated marker iNOS, whereas SP treatment lowered iNOS expression and up-regulated Arg1, an M2-associated marker (Fig. 3G to I). These findings suggested that SP not only reduced pro-inflammatory cytokine production but also promoted macrophage transition toward a repair-oriented phenotype.

To further investigate the mechanism underlying the immunomodulatory effects of SP, we used aprepitant to examine inflammatory cytokine secretion and polarization-related protein expression in RAW264.7 cells under different treatment conditions. Treatment with 10 μg/ml SP reduced LPS-induced IL-6 and TNF-α secretion. These inhibitory effects were markedly attenuated by aprepitant (Fig. S2B and C). Western blot analysis showed that SP decreased iNOS expression and increased Arg1 expression. Aprepitant attenuated both effects (Fig. S2D and E). These findings indicate that the immunomodulatory effects of SP are at least partially dependent on NK-1R. We also examined NK-1R-related downstream signaling proteins in RAW264.7 cells. Compared with the control group, LPS stimulation increased the p-AKT/AKT and p-ERK1/2/ERK1/2 levels. In LPS-stimulated cells, SP further increased the p-AKT/AKT level but reduced the p-ERK1/2/ERK1/2 level. Aprepitant markedly attenuated the effects of SP on AKT and ERK1/2 phosphorylation (Fig. S2F and G). These findings suggest that SP may enhance AKT signaling and suppress LPS-induced ERK1/2 activation through NK-1R. This signaling pattern may reduce the pro-inflammatory response of macrophages and promote a repair-associated phenotype.

Subsequently, the activity of free SP was evaluated using the CCK-8 assay. Free SP without plasma incubation increased cell viability at 5 μg/ml, with the greatest effect observed at 10 μg/ml (Fig. 3J). However, the activity of free SP decreased rapidly after incubation in plasma. After 60 min of plasma incubation, the pro-viability effect of SP was largely abolished (Fig. 3K). This limited stability restricted the direct use of soluble SP and suggested the need for a local delivery system capable of retaining and gradually releasing SP at the wound site.

### Synthesis, physicochemical characterization, and preliminary biocompatibility of the CS-SP-Nap-β@ZnO hydrogel

To enhance the local retention and functional stability of SP, a CS-based self-assembling hydrogel was prepared. CS, SP, and Nap-β served as the principal components, and CS-SP-Nap-β@ZnO was fabricated through an EDC/NHS-mediated amidation reaction (Fig. S3A). Briefly, SP was first grafted onto CS through the reaction between activated carboxyl groups and amino groups, followed by the grafting of Nap-β onto the CS-SP backbone. The chemical structure of the hydrogel was verified by FT-IR, UV–Vis, and ^1^H NMR assessments. In the FT-IR spectra, CS-SP-Nap-β demonstrated characteristic amide peaks at 1,543 and 1,629 cm^−1^, along with CS-related peaks, confirming the successful grafting of SP and Nap-β onto CS (Fig. 4A and Fig. S3B). UV–Vis spectra also demonstrated peptide-related absorption in the 190 to 204 nm range and a ZnO-related absorption peak about 372 nm after ZnO introduction (Fig. 4B). In addition, the ^1^H NMR spectrum demonstrated CS-derived proton signals at 3.0 to 4.0 parts per million (ppm) and aromatic proton signals about 7.13 ppm, further verifying the introduction of peptide components into the CS backbone (Fig. 4C). Standard curves were established based on the characteristic UV absorption peaks of SP and Nap-β (Fig. S3C and D). Quantitative analysis showed that SP exhibited a grafting efficiency of 61.18% and a degree of substitution of 0.0075 mol%, whereas the corresponding values for Nap-β were 46.46% and 0.0821 mol%, respectively (Table S6). The hydrogel-forming capacity was subsequently assessed. Tube-inversion tests indicated that CS-SP alone did not form a stable gel, whereas CS-SP-Nap-β generated a viscous hydrogel after Nap-β introduction. After ZnO incorporation, CS-SP-Nap-β@ZnO remained structurally stable without flowing upon inversion, indicating that ZnO further reinforced the hydrogel network (Fig. 4D). SEM images revealed an irregular but interconnected porous network in CS-SP-Nap-β, whereas ZnO incorporation resulted in a denser and more compact network morphology. TEM further indicated that ZnO nanoparticles were distributed within the hydrogel matrix, with particle sizes of about 30 to 40 nm (Fig. 4E). Macroscopic observations demonstrated that the CS-SP-Nap-β@ZnO hydrogel exhibited good self-healing ability, injectability, and adhesive properties (Fig. S4A). Mechanical testing showed that the incorporation of ZnO nanoparticles did not substantially affect the tensile properties of the hydrogel (Fig. S4B and C). However, compared with CS-SP-Nap-β, the CS-SP-Nap-β@ZnO hydrogel exhibited higher compressive load-bearing capacity and compressive Young’s modulus (Fig. S4D and E). These results indicate that ZnO nanoparticles reinforced the hydrogel network while maintaining its favorable deformability.

**Fig. 4.**
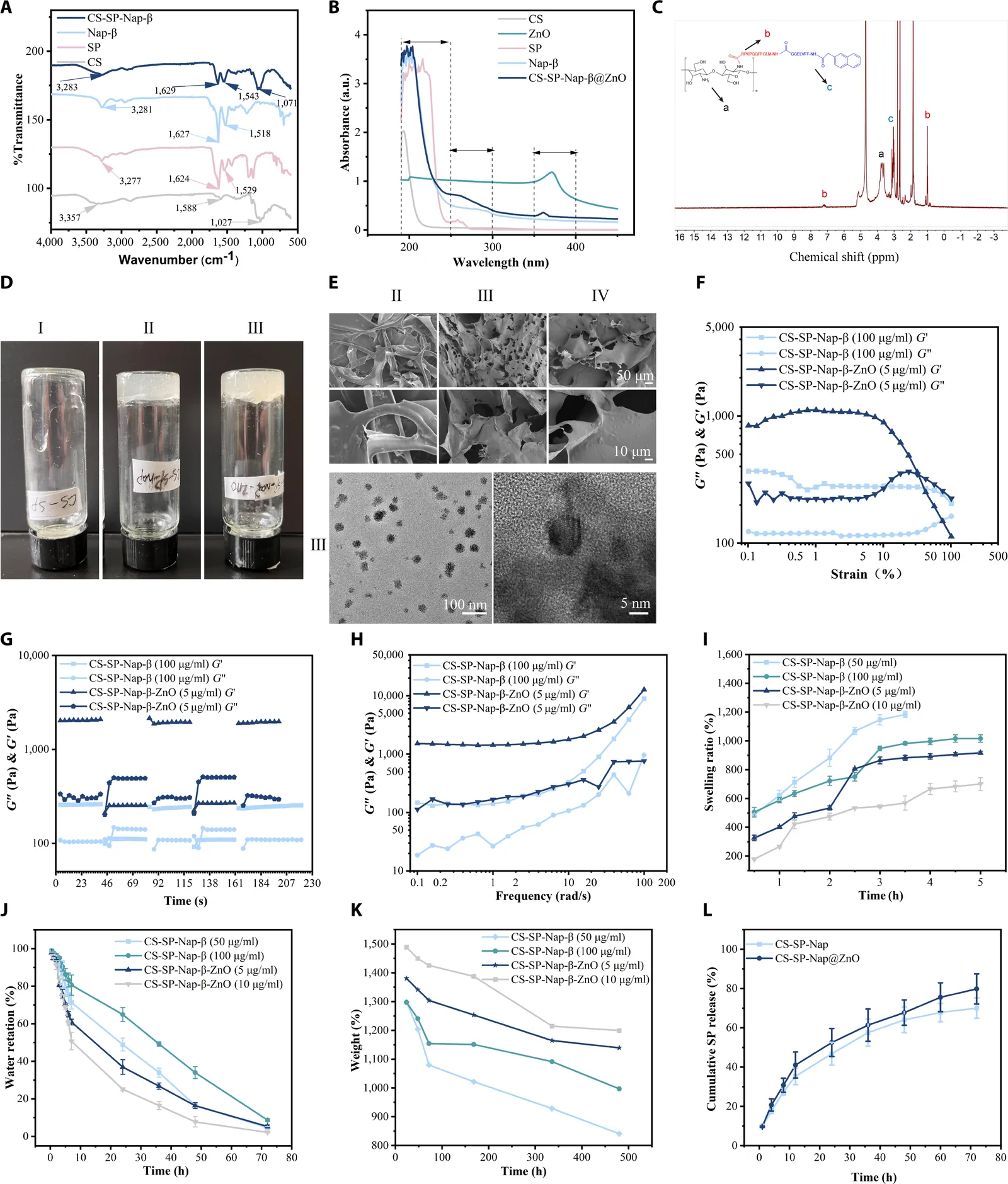
Synthesis and physicochemical characterization of the CS-SP-Nap-β@ZnO hydrogel. (A) FT-IR spectra of CS, SP, Nap-β, and CS-SP-Nap-β. (B) UV–Vis spectra of CS, SP, Nap-β, ZnO, CS-SP-Nap-β, and CS-SP-Nap-β@ZnO. (C) ^1^H NMR spectrum of CS-SP-Nap-β. (D) Tube-inversion photographs. I: CS-SP, II: CS-SP-Nap-β, III: CS-SP-Nap-β@ZnO. (E) SEM and TEM images showing hydrogel morphology. Scale bars are indicated in the corresponding images. II: CS-SP-Nap-β, III: CS-SP-Nap-β@ZnO (5 μg/ml), IV: CS-SP-Nap-β@ZnO (10 μg/ml) (F to H) Rheological characterization of CS-SP-Nap-β and CS-SP-Nap-β@ZnO hydrogels, including amplitude-sweep, self-healing, and frequency-sweep tests. (I to K) Swelling ratio, water-retention capacity, and degradation profiles of different hydrogel formulations. (L) Cumulative release of SP from CS-SP-Nap-β and CS-SP-Nap-β@ZnO hydrogels during 72 h. Data are expressed as the mean ± SD (*n* = 3).

Rheological assessment further verified the formation of a stable hydrogel network. Time-sweep measurements showed that CS-SP-Nap-β reached the rheological gel point at approximately 50 min (Fig. S4F). Amplitude sweep tests suggested that the optimized CS-SP-Nap-β@ZnO formulation retained a stable viscoelastic region, whereas excessive ZnO loading lowered structural stability under high strain (Fig. 4F and Fig. S4G). Self-healing and frequency sweep tests indicated that CS-SP-Nap-β@ZnO recovered after repeated structural disruption and retained *G*′ above *G*″ over the tested frequency range, confirming stable gel-like behavior (Fig. 4G and H). The dressing-related properties of the hydrogel were then assessed. The optimized CS-SP-Nap-β@ZnO hydrogel demonstrated a stable swelling behavior, favorable water-retention potential, and gradual degradation, indicating its suitability for maintaining a moist wound microenvironment while allowing gradual material remodeling (Fig. 4I to K). SP release tests demonstrated continuous SP release from both CS-SP-Nap-β and CS-SP-Nap-β@ZnO over 72 h, and ZnO introduction did not prevent peptide release from the hydrogel network (Fig. 4L). Subsequently, the hydrogel eluate was collected after 72 h of incubation to evaluate the bioactivity of SP following release. The CS-SP-Nap-β@ZnO hydrogel eluate continued to enhance cell viability, indicating that the released SP retained its biological activity (Fig. S5A). We further evaluated the release behavior of ZnO from CS-SP-Nap-β@ZnO. ZnO was gradually released from the hydrogel, reaching a cumulative release of approximately 66.4% at 72 h (Fig. S5B). Furthermore, the soluble Zn^2+^ concentration in the release medium reached approximately 11.37 μmol/L at 72 h (Fig. S5C).

Finally, the preliminary biological compatibility of the hydrogel was assessed. CCK-8 tests indicated that HSFs and HaCaT cells retained favorable viability after treatment with different hydrogel components (Fig. S6A and B). Live/dead staining also verified that HSFs and HaCaT cells remained viable after coculture with CS-Nap-β, CS-SP-Nap-β, and CS-SP-Nap-β@ZnO, with only a small number of PI-positive dead cells observed (Fig. S6C and D). Hemolysis tests demonstrated no obvious hemolysis after incubation with SP, Nap-β, ZnO, or CS-SP-Nap-β@ZnO, and the hemolysis rates of all formulation groups stayed below 5% (Fig. S6E). These findings suggested that CS-SP-Nap-β@ZnO had appropriate structural stability, sustained SP-release behavior, and favorable cytocompatibility and hemocompatibility for subsequent biological experiments.

### CS-SP-Nap-β@ZnO hydrogel promotes macrophage transition toward a reparative phenotype

Persistent macrophage-mediated inflammation is a central feature of chronic wounds. Accordingly, we first assessed whether the hydrogel could regulate macrophage pro-inflammatory activation. LPS stimulation elevated TNF-α and IL-6 production, whereas SP, CS-SP-Nap-β, and CS-SP-Nap-β@ZnO markedly lowered the levels of these cytokines. Among these treatments, CS-SP-Nap-β@ZnO demonstrated the strongest inhibitory response (Fig. 5A and B).

**Fig. 5.**
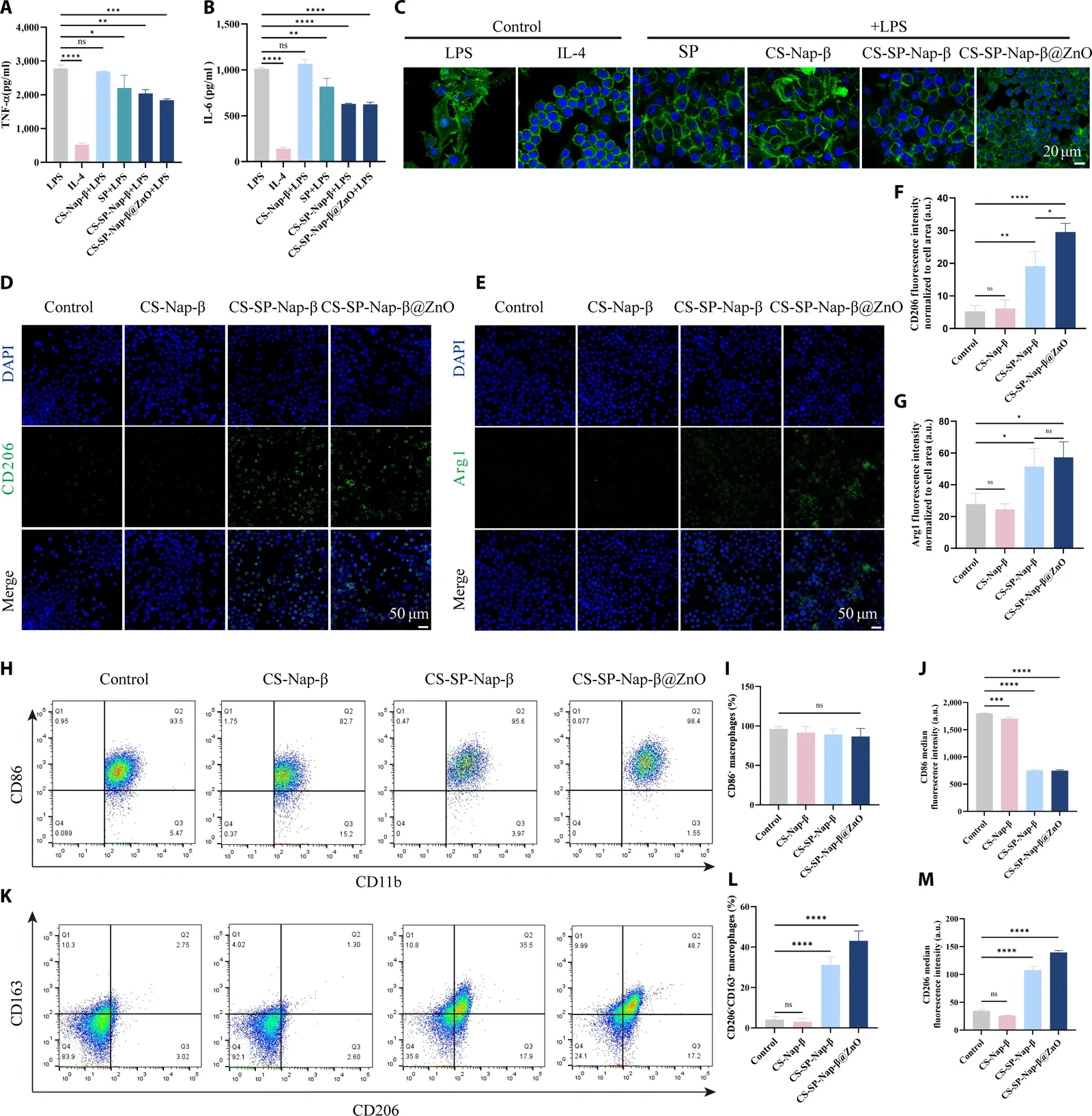
Macrophage phenotype regulation by the CS-SP-Nap-β@ZnO hydrogel. (A and B) TNF-α and IL-6 levels in LPS-challenged macrophages after the indicated treatments. (C) F-actin cytoskeletal staining of RAW264.7 macrophages after LPS stimulation and treatment with IL-4, SP, CS-Nap-β, CS-SP-Nap-β, or CS-SP-Nap-β@ZnO. Scale bar, 20 μm. (D) Immunofluorescence staining for CD206. Scale bar, 50 μm. (E) Immunofluorescence staining for Arg1. Scale bar, 50 μm. (F and G) Quantification of CD206 and Arg1 fluorescence intensity. (H) Representative flow cytometry plots of CD11b and CD86 expression in LPS-stimulated macrophages following different treatments. (I) Quantification of the proportion of CD86^+^ macrophages. (J) Quantification of CD86 median fluorescence intensity. (K) Representative flow cytometry plots of CD206 and CD163 expression in LPS-stimulated macrophages following different treatments. (L) Quantification of the proportion of CD206^+^CD163^+^ macrophages. (M) Quantification of CD206 median fluorescence intensity. Data are expressed as the mean ± SD (*n* = 3). ns, not statistically significant; *P < 0.05, **P < 0.01, ***P < 0.001, ****P < 0.0001.

Morphological alterations in RAW264.7 cells were subsequently assessed by cytoskeletal staining. LPS-exposed macrophages demonstrated an activated pro-inflammatory morphology, whereas IL-4-treated macrophages showed a smoother and more regular morphology. Following treatment with SP-containing hydrogels, especially CS-SP-Nap-β@ZnO, the cytoskeletal morphology shifted toward that of IL-4-treated cells (Fig. 5C).

Immunofluorescence staining also indicated that CS-SP-Nap-β and CS-SP-Nap-β@ZnO up-regulated CD206 and Arg1, whereas CS-Nap-β alone demonstrated only modest responses (Fig. 5D to G). Flow cytometry was subsequently conducted to further assess macrophage phenotype modulation. Representative gating plots demonstrated the sequential identification of CD45^+^ immune cells and CD45^+^CD11b^+^F4/80^+^ macrophages (Fig. S7A and B). Under LPS stimulation, hydrogels containing SP reduced CD86 median fluorescence intensity without markedly altering the proportion of CD86^+^ macrophages (Fig. 5H to J). Meanwhile, both CS-SP-Nap-β and CS-SP-Nap-β@ZnO increased the proportion of CD206^+^CD163^+^ macrophages and CD206 median fluorescence intensity, with CS-SP-Nap-β@ZnO showing the greatest effects (Fig. 5K to M). These findings suggest that CS-SP-Nap-β@ZnO attenuates CD86 expression while promoting the acquisition of a reparative macrophage phenotype, rather than inducing a complete loss of the CD86-positive population.

### CS-SP-Nap-β@ZnO hydrogel attenuates oxidative damage and supports repair-related cell proliferation and migration in vitro

Oxidative stress is another central factor that maintains chronic inflammation and impedes wound healing. ROS scavenging assays conducted in a cell-free system showed that ZnO nanoparticles directly reduced O_2_•^−^ and H_2_O_2_ levels. At 5 μg/ml, the scavenging efficiencies for O_2_•^−^ and H_2_O_2_ were 45.3% and 46.6%, respectively (Fig. S7C and D), demonstrating the ability of ZnO nanoparticles to remove these 2 ROS species. Accordingly, we examined the antioxidant response of CS-SP-Nap-β@ZnO under H_2_O_2_-induced oxidative damage. DCFH-DA staining demonstrated intense intracellular green fluorescence after H_2_O_2_ treatment, indicating pronounced intracellular ROS accumulation. Pretreatment with CS-SP-Nap-β@ZnO lowered the DCFH-DA fluorescence signal, whereas CS-Nap-β demonstrated a more limited protective effect. Quantitative assessment also verified the reduction in ROS-positive cells after CS-SP-Nap-β@ZnO treatment (Fig. 6A and B). Flow cytometric analysis also indicated that CS-SP-Nap-β@ZnO reduced intracellular ROS levels under H_2_O_2_ stimulation (Fig. 6C). Consistently, Annexin V-FITC/PI staining indicated that H_2_O_2_ elevated cellular apoptosis, whereas CS-SP-Nap-β@ZnO lowered H_2_O_2_-induced apoptosis (Fig. 6D).

**Fig. 6.**
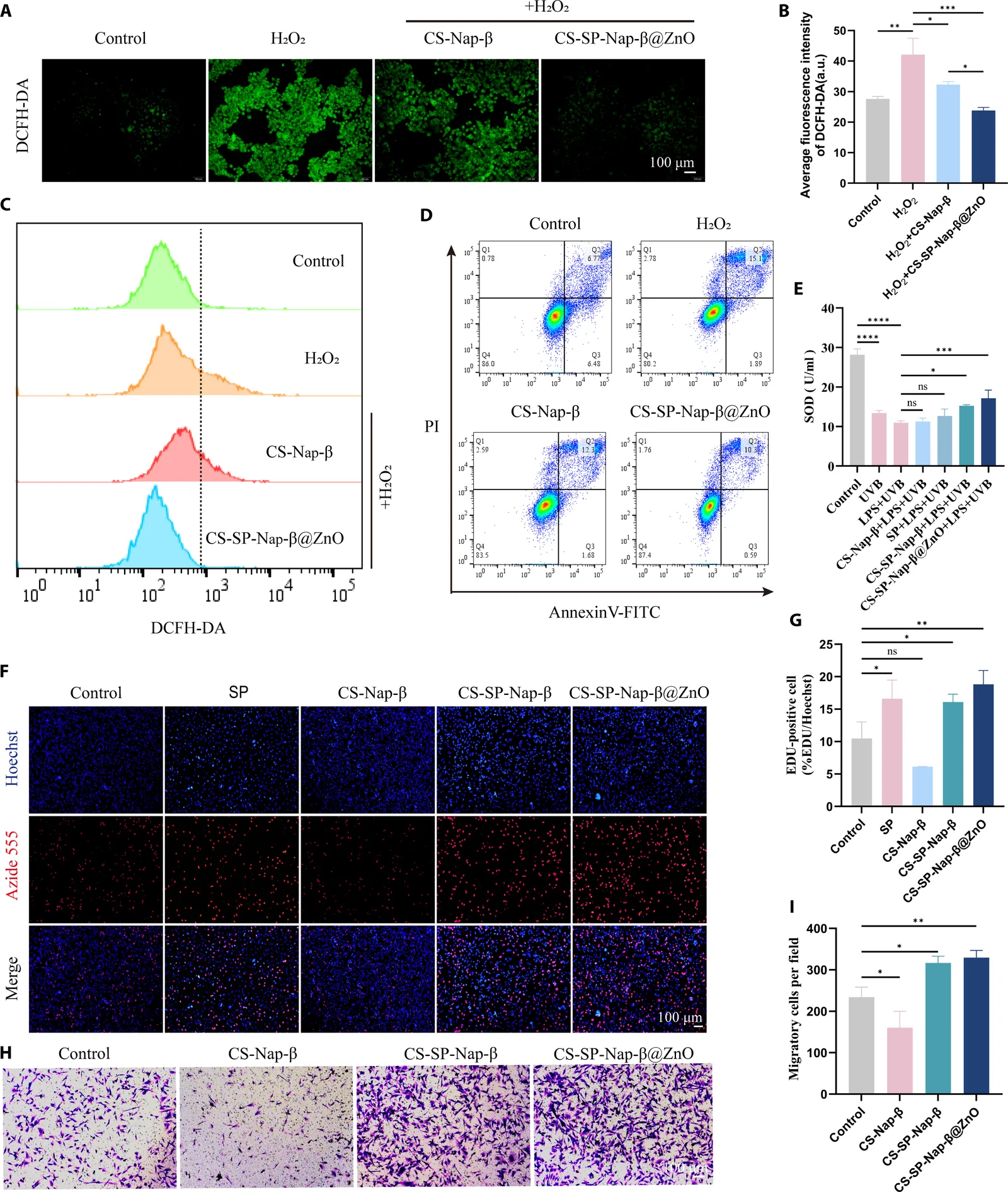
Antioxidant activity and in vitro wound-healing effects of the CS-SP-Nap-β@ZnO hydrogel. (A) DCFH-DA staining showing intracellular ROS in H_2_O_2_-challenged cells after the indicated treatments. Scale bar, 100 μm. (B) Quantitative analysis of ROS-positive cell populations. (C) Flow cytometric analysis of intracellular ROS levels. (D) Annexin V-FITC/PI staining-based analysis of apoptosis. (E) Intracellular SOD activity in macrophages after combined UVB and LPS stimulation. (F and G) Representative EdU staining images and quantification of cell proliferation. Scale bar, 100 μm. (H and I) Representative Transwell images and quantification of migrated cells. Scale bar, 100 μm. Data are expressed as the mean ± SD (*n* = 3). ns, not statistically significant; *P < 0.05, **P < 0.01, ***P < 0.001, ****P < 0.0001.

To also evaluate antioxidant activity in an inflammation-related oxidative microenvironment, macrophages were exposed to UVB irradiation and LPS stimulation, and intracellular SOD activity was quantified. UVB and LPS lowered SOD activity, whereas SP-containing hydrogel treatment partially restored SOD activity. Among all groups, CS-SP-Nap-β@ZnO demonstrated a more pronounced SOD recovery response, indicating that ZnO introduction enhanced the antioxidant potential of the hydrogel (Fig. 6E).

Given that cellular proliferation and migration are essential for wound contraction, we subsequently evaluated whether the hydrogel could support these processes. EdU staining indicated that SP, CS-SP-Nap-β, and CS-SP-Nap-β@ZnO elevated the proportion of proliferating cells relative to the untreated control and CS-Nap-β groups. The CS-SP-Nap-β@ZnO group demonstrated the highest EdU-positive cell ratio (Fig. 6F and G). In the Transwell migration test, CS-SP-Nap-β and CS-SP-Nap-β@ZnO elevated the proportion of migrated cells, whereas CS-Nap-β alone demonstrated a modest response on cellular migration (Fig. 6H and I). To determine whether these effects also extended to keratinocytes, we performed validation experiments using HaCaT cells and found that CS-SP-Nap-β@ZnO similarly promoted cell proliferation and migration (Fig. S7E to H). These results indicated that CS-SP-Nap-β@ZnO reduced oxidative injury while supporting the proliferation and migration of wound-related cells.

### CS-SP-Nap-β@ZnO hydrogel promotes wound closure in a UVB-induced delayed-healing wound model

On the basis of the cellular findings, we also assessed the therapeutic efficacy of the hydrogel in a UVB-induced delayed-healing model system. Rats were exposed to UVB irradiation before full-thickness wound creation, and the wounds were treated with control solution, CS-Nap-β, free SP, CS-SP-Nap-β, or CS-SP-Nap-β@ZnO (Fig. 7A). Before assessing treatment outcomes, the UVB-induced skin injury model was first verified. Macroscopic observation demonstrated visible dorsal skin damage after UVB irradiation (Fig. S8A). H&E and Masson’s staining also demonstrated epidermal thickening, disrupted skin structure, and pro-inflammatory changes after UVB exposure and wound creation, confirming the successful establishment of a UVB-induced delayed-healing model (Fig. S8B).

**Fig. 7.**
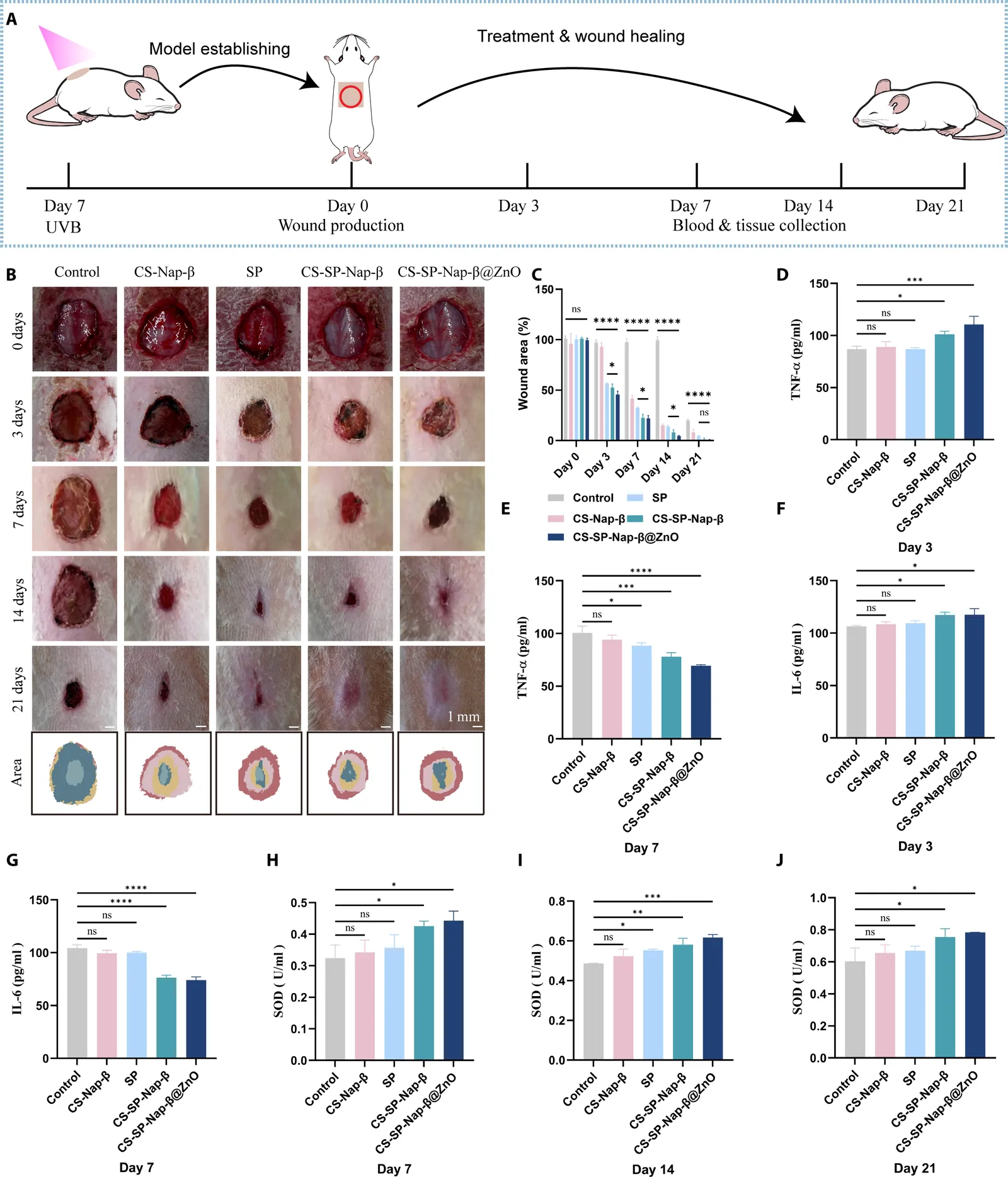
CS-SP-Nap-β@ZnO hydrogel accelerates wound closure in a UVB-induced delayed-healing wound model. (A) Schematic illustration of the animal experimental workflow. (B) Representative wound images after the indicated treatments on days 0, 3, 7, 14, and 21. Scale bar, 1 mm. (C) Quantification of residual wound area. (D to G) Serum TNF-α and IL-6 levels on days 3 and 7 after treatment. (H to J) Serum SOD activity on days 7, 14, and 21 after treatment. Data are expressed as the mean ± SD (*n* = 3 rats per group). ns, not statistically significant; *P < 0.05, **P < 0.01, ***P < 0.001, ****P < 0.0001.

Representative wound images showed faster wound closure in all SP-containing treatment groups. Compared with free SP treatment, the CS-SP-Nap-β@ZnO group exhibited markedly smaller residual wound areas on days 3, 7, and 14. Moreover, the residual wound area in the CS-SP-Nap-β@ZnO group was substantially smaller than that in the untreated control group at all posttreatment time points (Fig. 7B and C). These results indicate that hydrogel-mediated sustained delivery of SP accelerates wound closure, particularly during the early and middle stages of healing.

Next, we further evaluated the regulation of systemic inflammatory responses during wound healing. On day 3, serum TNF-α and IL-6 levels were markedly higher in the CS-SP-Nap-β and CS-SP-Nap-β@ZnO groups than in the untreated control group, whereas no substantial differences were observed between the free SP and untreated control groups. By day 7, the levels of both inflammatory cytokines were significantly lower in the CS-SP-Nap-β and CS-SP-Nap-β@ZnO groups than in the untreated control group. In contrast, free SP alone exerted only a limited modulatory effect on serum inflammatory cytokine levels, with a substantial reduction observed only in TNF-α on day 7 (Fig. 7D to G). Measurements on days 14 and 21 showed a continued downward trend in serum TNF-α and IL-6 levels in the CS-SP-Nap-β@ZnO group, consistent with the gradual resolution of inflammation during the later stages of wound healing (Fig. S8C to F).

Serum SOD activity was also measured to assess systemic antioxidant status. On day 3, no marked differences in serum SOD activity were observed among the treatment groups compared with the untreated control group (Fig. S8G). At the subsequent time points, both the CS-SP-Nap-β and CS-SP-Nap-β@ZnO groups exhibited higher SOD activity, with the CS-SP-Nap-β@ZnO group showing the highest activity (Fig. 7H to J). These findings indicate that CS-SP-Nap-β@ZnO enhances systemic antioxidant defense. Collectively, these results suggest that CS-SP-Nap-β@ZnO accelerates wound contraction by coordinating inflammatory regulation with antioxidant protection.

### CS-SP-Nap-β@ZnO hydrogel supports re-epithelialization, collagen deposition, and angiogenesis

Histological evaluation was performed to further assess wound repair. H&E staining on days 3 and 7 revealed inflammatory cell infiltration in all groups, which appeared less extensive in the CS-SP-Nap-β@ZnO group (Fig. 8A). Masson’s trichrome staining showed more pronounced collagen deposition in this group on day 7 (Fig. 8B). On day 21, H&E staining showed that large areas of the wound remained incompletely remodeled in the control group. In contrast, the SP-containing hydrogel groups exhibited more complete re-epithelialization and better-organized tissue architecture, with the CS-SP-Nap-β@ZnO group showing the smallest incompletely remodeled area (Fig. 8C and D). Masson’s trichrome staining and quantitative analysis further revealed denser, more regularly organized collagen fibers and the highest collagen volume fraction in this group (Fig. 8C and E).

**Fig. 8.**
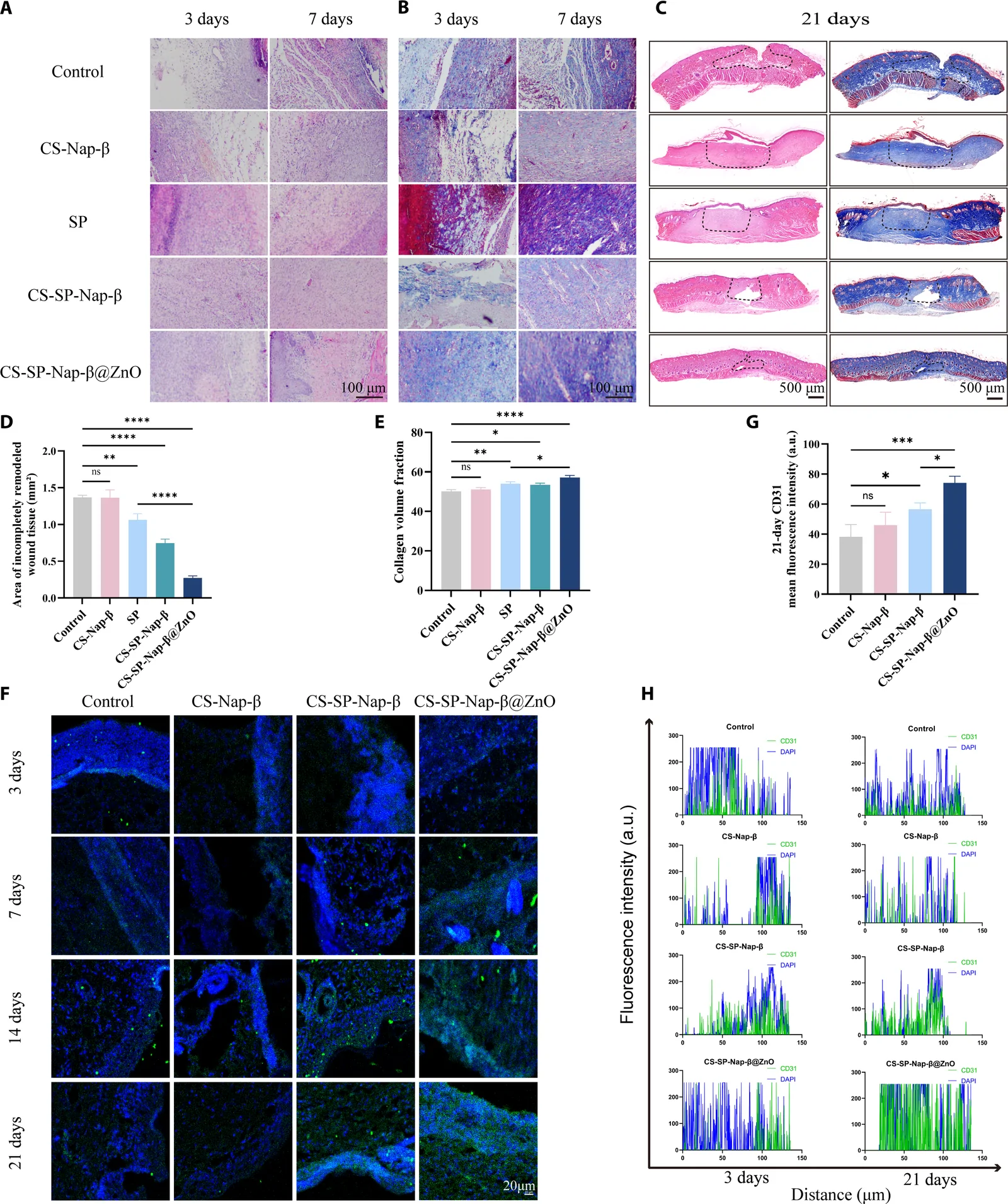
CS-SP-Nap-β@ZnO hydrogel enhances tissue regeneration during wound repair. (A and B) Representative H&E (A) and Masson’s trichrome (B) staining of wound tissues on days 3 and 7. Scale bar, 100 μm. (C) Representative H&E (left) and Masson’s trichrome (right) staining on day 21. Dashed outlines indicate incompletely remodeled wound regions. Scale bars, 500 μm. (D) Quantification of the incompletely remodeled wound area on day 21. (E) Quantification of the collagen volume fraction on day 21. (F) Representative immunofluorescence images of CD31 (green) and DAPI (blue) on days 3, 7, 14, and 21. Scale bar, 20 μm. (G) Quantification of CD31 fluorescence intensity on day 21. (H) Representative fluorescence intensity profiles of CD31 and DAPI across wound sections on days 3 and 21. Data are expressed as the mean ± SD (*n* = 3 rats per group). ns, not statistically significant; *P < 0.05, **P < 0.01, ***P < 0.001, ****P < 0.0001.

Immunofluorescence staining showed that SP-containing hydrogels markedly enhanced CD31-positive signals in the wound tissue. On day 21, the CS-SP-Nap-β@ZnO group exhibited the strongest and most broadly distributed CD31 fluorescence, with an intensity markedly higher than that of the control group (Fig. 8F to H). Further analysis showed that hydrogel treatment altered the local expression of markers associated with immune phenotypes in the wound tissue (Fig. 9A). No significant differences in CD86 or CD206 expression were observed between the control and CS-Nap-β groups. In contrast, treatment with hydrogels containing SP decreased CD86 expression and increased CD206 expression, with the most pronounced changes observed in the CS-SP-Nap-β@ZnO group (Fig. 9B and C). This finding was consistent with an improved reparative immune microenvironment. Together with the in vitro flow cytometry results obtained from F4/80^+^ macrophages, these findings support the immunomodulatory effect of the hydrogel on the local immune phenotype. To further evaluate the in vivo biosafety of CS-SP-Nap-β@ZnO, serum ALT, urea, and Zn^2+^ levels were measured in all groups after 21 days of treatment. No significant differences were observed among the groups (Fig. S8H to J). In addition, H&E staining of major organs demonstrated no apparent pathological abnormalities after treatment, suggesting preliminary in vivo safety of the hydrogel platform (Fig. S9). Taken together, these histological observations indicated that CS-SP-Nap-β@ZnO enhanced wound healing by enhancing re-epithelialization, collagen remodeling, and angiogenesis.

**Fig. 9.**
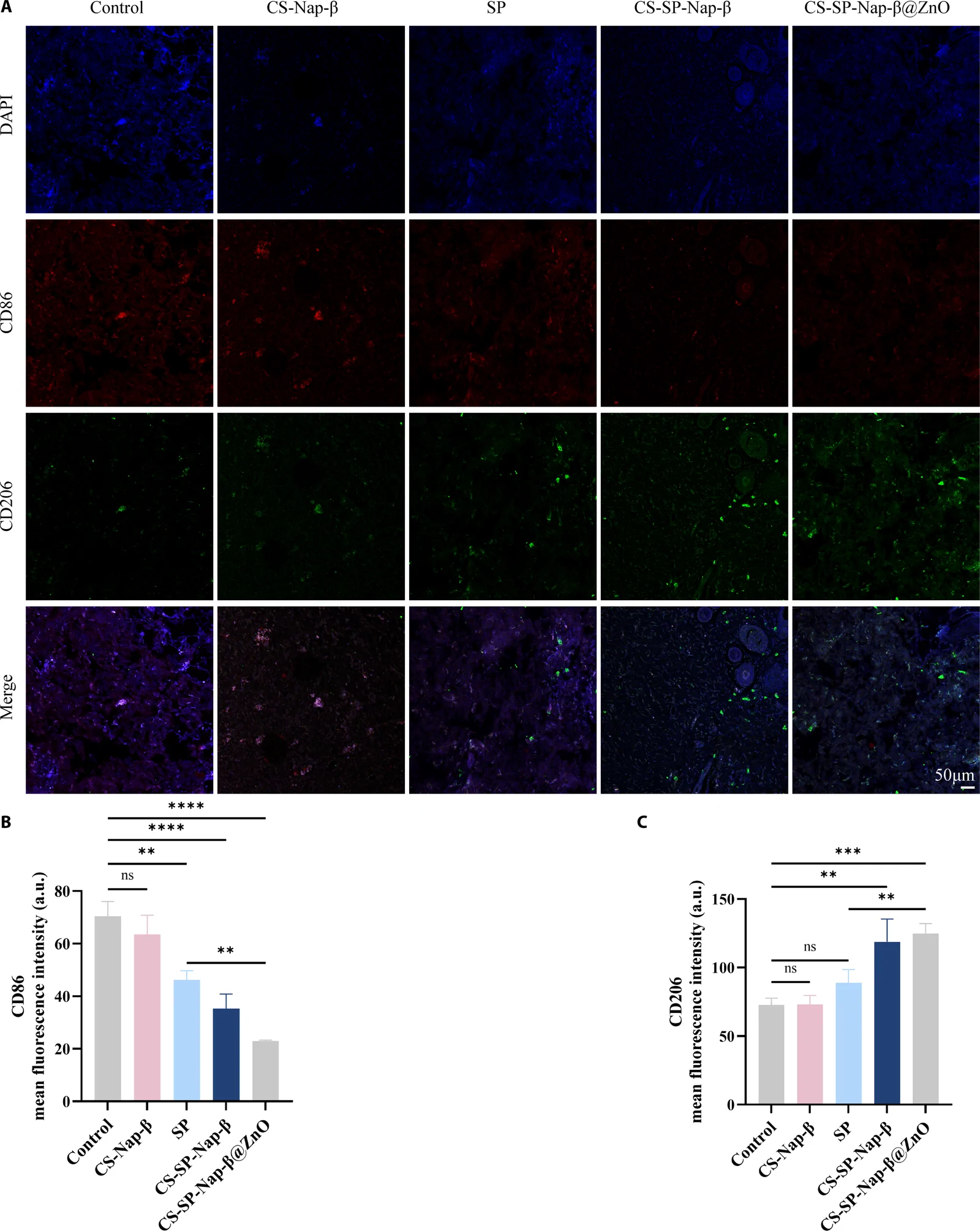
CS-SP-Nap-β@ZnO hydrogel promotes remodeling of the local immune phenotype in wound tissues. (A) Representative immunofluorescence images of CD86 (red), CD206 (green), and DAPI (blue). Scale bar, 50 μm. (B and C) Quantification of the mean fluorescence intensities of CD86 (B) and CD206 (C). Data are expressed as the mean ± SD (*n* = 3 rats per group). ns, not statistically significant; *P < 0.05, **P < 0.01, ***P < 0.001, ****P < 0.0001.

## Discussion and Conclusion

Chronic wound repair is often limited by persistent inflammation, excessive oxidative stress, and impaired activity of wound-related cells [2,42]. These pathological features are closely interrelated: pro-inflammatory activation supports intracellular ROS accumulation, whereas oxidative stress also worsens inflammatory injury and delays tissue remodeling. Accordingly, a wound dressing that provides only passive coverage is likely insufficient for delayed wound healing [37].

In recent years, wound dressing design has increasingly shifted toward multifunctional nanoengineered hydrogel platforms that coordinate multiple stages of wound repair by integrating pro-angiogenic, wound microenvironment-responsive, redox-regulatory, and immunomodulatory functions [43]. Meanwhile, therapeutic peptides have been increasingly applied in tissue repair and wound healing because of their potent and specific biological activities [44]. In this study, we designed a CS-SP-Nap-β@ZnO hydrogel to address these 2 interrelated barriers by combining sustained SP delivery with ZnO-assisted antioxidant activity.

SP was chosen because it is closely associated with neuroimmune modulation and tissue regeneration [14,45]. Our bioinformatics assessment revealed TAC1/SP as a secreted candidate associated with wound healing, and molecular docking indicated a possible interaction between SP and NK-1R. Functionally, SP supported endothelial cellular migration and lowered pro-inflammatory cytokine release in LPS-stimulated macrophages. SP also reduced iNOS expression and elevated Arg1 expression, indicating that SP may help guide macrophages away from an inflammatory state. These findings are in agreement with previous studies indicating that SP participates in immune cell recruitment, epithelial healing, vascular response, and tissue repair [10,12,14,46]. However, free SP rapidly lost activity after incubation in plasma, indicating that direct topical administration may not maintain a sufficient local concentration at the wound site. This finding provided the basis for incorporating SP into a local delivery hydrogel.

The CS-SP-Nap-β hydrogel was prepared to enhance SP retention and release. CS was employed as the primary polymer backbone because of its biocompatibility, biodegradability, and chemically reactive amino groups [34,35]. Nap-β was introduced to promote self-assembly and hydrogel network formation. The materials characterization findings verified the successful formation of the CS-SP-Nap-β network and the introduction of ZnO. Tube-inversion, SEM/TEM, rheological, swelling, degradation, and release tests indicated that CS-SP-Nap-β@ZnO generated a stable porous hydrogel with self-healing behavior, water-retention potential, gradual degradation, and sustained release of SP-related components. These properties are notable for wound dressings because they help preserve a moist local microenvironment whereas permitting continuous exposure of the wound bed to the bioactive peptide [37].

The incorporation of ZnO nanoparticles further enhanced the antioxidant properties of the hydrogel [37–39]. In our cell-free assays, 5 μg/ml ZnO nanoparticles reduced O_2_•^−^ and H_2_O_2_ by 45.3% and 46.6%, respectively. The activity observed at this comparatively low concentration may be associated with the large surface availability of the nanoparticles, which facilitates ROS adsorption and surface electron transfer reactions [47]. Excessive ROS represents a central factor that damages keratinocytes, fibroblasts, endothelial cells, and macrophages during delayed wound healing. In this study, CS-SP-Nap-β@ZnO lowered intracellular ROS accumulation in H_2_O_2_-exposed cells and reduced oxidative damage-related apoptosis. In an inflammation-associated oxidative model system induced by UVB and LPS, the hydrogel also partially restored intracellular SOD activity. The stronger antioxidant response observed after ZnO incorporation suggests that ZnO contributed to the protective effect. Instead of relying on a rigid conventional MOF structure, the present design employed ZnO within a CS-peptide hydrogel to provide a hydrated antioxidant environment suitable for local wound treatment. This strategy may be better suited for local wound treatment, where flexibility, hydration, degradability, and biocompatibility are notable.

Macrophage modulation was another notable function of this formulation. Persistent macrophage-mediated inflammation is a central feature of chronic and delayed-healing wounds [4,48]. In vitro, CS-SP-Nap-β@ZnO lowered TNF-α and IL-6 production, changed macrophage morphology, elevated CD206 and Arg1 expression, and expanded CD206/CD163-positive macrophage populations. These observations indicate that the hydrogel did more than simply suppress inflammation but also substantiated the transition toward a reparative macrophage phenotype. This response may be associated with sustained release of SP-related components, whereas the antioxidant effect of ZnO may also reduce ROS-driven pro-inflammatory activation. Accordingly, the material appears to function through combined effects on inflammatory activation and oxidative stress.

The in vivo findings also substantiated the wound-healing potential of CS-SP-Nap-β@ZnO. In the UVB-induced delayed-healing model system, hydrogel treatment hastened wound contraction relative to the untreated control and CS-Nap-β groups. Serum pro-inflammatory cytokines declined more rapidly throughout the healing phase, and SOD activity increased, particularly in the CS-SP-Nap-β@ZnO group. Histological evaluation demonstrated enhanced re-epithelialization, collagen deposition, and CD31-positive neovascularization. These findings are in agreement with the cellular observations, indicating that the hydrogel enhanced the local wound microenvironment and supported several stages of tissue regeneration. The enhancement in re-epithelialization and angiogenesis may be linked to SP-mediated cellular migration and ZnO-assisted oxidative stress control, whereas the improved collagen deposition may reflect a more favorable regenerative microenvironment during the later healing phase.

Several limitations should be acknowledged. First, the UVB-induced delayed-healing model system reflects photodamage-associated oxidative and pro-inflammatory injury, but it does not fully recapitulate all features of clinical chronic wounds including diabetes, infection, ischemia, and pressure-related injury [2,49]. In addition, since this model does not include a non-UVB full-thickness excision group, it is not possible to quantify the independent effect of UVB pretreatment on delayed wound healing. Accordingly, the therapeutic performance of CS-SP-Nap-β@ZnO should also be assessed in other clinically relevant wound models. Second, although wound-tissue CD86/CD206 staining, serum inflammatory cytokines, SOD activity, and regenerative outcomes support macrophage modulation and antioxidant effects in vivo, the underlying mechanisms remain unclear. Further wound-site oxidative stress analyses and pathway-specific interventions are needed to clarify the links among immune regulation, redox balance, and tissue repair. Third, the long-term degradation behavior and zinc-related metabolic safety of the hydrogel require additional investigation before translational application.

In this study, we developed a CS-SP-Nap-β@ZnO hydrogel that combines local neuropeptide delivery with ZnO-assisted antioxidant activity. The CS–peptide network supported hydrogel formation, self-healing behavior, water retention, gradual degradation, and sustained release of SP-related components, while maintaining acceptable cytocompatibility and hemocompatibility. In vitro, CS-SP-Nap-β@ZnO reduced inflammatory cytokine production, promoted macrophage transition toward a reparative phenotype, decreased ROS accumulation, and supported the proliferation and migration of wound-related cells. In a UVB-induced delayed-healing wound model, the hydrogel accelerated wound closure and improved re-epithelialization, collagen deposition, and neovascularization. These findings support the potential of CS-SP-Nap-β@ZnO as a local dressing for wound repair following UVB-induced skin injury.

## Data Availability

The authors confirm that the data supporting the findings of this study are available within the article.
