# Supplementary material for: A Chitosan-Based Neuropeptide Hydrogel with Zinc Oxide-Assisted Antioxidant Activity for Ultraviolet B-Induced Delayed Wound Repair
Source: Biomater Res. 2026 Nov 9;30:0443. doi: 10.34133/bmr.0443 (PMC13745762; doi:10.34133/bmr.0443)
Supplement: Supplementary file 1 [file bmr.0443.f1.docx]

**Supporting information**

**A chitosan-based neuropeptide hydrogel with ZnO-assisted antioxidant activity for UVB-induced delayed wound repair**

Hanying Wang^1, 2, †^, Qian Chen^2, †^, Guizhu Feng^6, †^, Qiqi Pei^2^, Jingpei Shi^1^, Yu Zhang^1^, Chengjie Ma^4^, Yubin Hu^5^, Shiwei Niu^3,^ *, Lixian Mu^2,^ *, Lechun Lv^1,^ *

**Affiliations**

1 Yunnan Key Laboratory of Plateau Thermal Medical Rehabilitation and Wellness, School of Rehabilitation, Kunming Medical University, Kunming, 650500, Yunnan, China.

2 School of Basic Medical Sciences, Kunming Medical University, Kunming, 650500, Yunnan, China.

3 Yunnan Key Laboratory of Stem Cell and Regenerative Medicine, College of Rehabilitation, Kunming Medical University, Kunming, 650500, PR China.

4 Department of Orthopedics, The First People’s Hospital of Yunnan Province & the Affiliated Hospital of Kunming University of Science and Technology, Kunming, 650500, Yunnan, China.

5 Department of Medical Imaging, First Affiliated Hospital of Kunming Medical University, Kunming, 650500, Yunnan, China.

6 School of Nursing，Kunming Medical University Kunming, 650500, Yunnan, China.

*Lechun Lv

E-mail: lvlechun@kmmu.edu.cn

*Lixian Mu

E-mail: Mulixian77@163.com

*Shiwei Niu

E-mail: niushiwei@kmmu.edu.cn

† These authors contributed equally to this work.

Supplementary Figure 1

Figure S1. Supplementary bioinformatic analysis of SP screening. (a-c) Differential gene expression analysis of wound-related datasets. (d) Predicted enzymatic cleavage sites and potential active fragments of SP.

Supplementary Figure 2

Figure S2. Role of NK-1R in the biological effects of SP. (a) Cell viability in different treatment groups measured using the CCK-8 assay. (b, c) IL-6 and TNF-α levels in RAW264.7 cell culture supernatants measured by ELISA. (d) Western blot analysis of iNOS and Arg1 protein expression. (e) Densitometric quantification of iNOS and Arg1 expression relative to GAPDH. (f) Western blot analysis of NK-1R, AKT, p-AKT, ERK1/2, and p-ERK1/2. (g) Densitometric quantification of p-AKT/AKT and p-ERK1/2/ERK1/2 ratios. Data are presented as the mean ± SD (n = 3 independent experiments). ns, not statistically significant; *P < 0.05, **P < 0.01, ***P < 0.001, and ****P < 0.0001.

Supplementary Figure 3

Figure S3. Supplementary characterization of CS-SP-Nap-β@ZnO hydrogel. (a) Schematic illustration of CS-SP-Nap-β@ZnO synthesis. (b) FT-IR spectra of CS-SP and CS-Nap-β. (c, d) Calibration curves of SP at 258 nm (c) and Nap-β at 278 nm (d). Data are presented as mean ± SD (n = 3).

Supplementary Figure 4

Figure S4. Macroscopic, mechanical, and rheological properties of the CS-SP-Nap-β@ZnO hydrogel. (a) Photographs demonstrating self-healing, injectability, shape adaptability, and adhesion. (b, c) Tensile stress–strain curves and corresponding Young’s moduli. (d, e) Compressive stress–strain curves and corresponding Young’s moduli. (f) Time-sweep rheological analysis of CS-SP-Nap-β. (g) Amplitude sweep analysis of different hydrogel formulations. Data are presented as the mean ± SD (n = 3 independent experiments). ns, not statistically significant; *P < 0.05, **P < 0.01, ***P < 0.001, and ****P < 0.0001.

Supplementary Figure 5

Figure S5. Bioactivity of SP released from the CS-SP-Nap-β@ZnO hydrogel and the release profiles of ZnO and Zn²⁺. (a) Cell viability following treatment with free SP or hydrogel eluates collected after 72 h of incubation. (b) Cumulative release of ZnO from the hydrogel. (c) Zn²⁺ concentration in the hydrogel release medium. Data are presented as the mean ± SD (n = 3 independent experiments). ns, not statistically significant; *p < 0.05, **p < 0.01, ***p < 0.001, and ****p < 0.0001.

Supplementary Figure 6

Figure S6. Preliminary biocompatibility evaluation of the hydrogel formulations. (a, b) CCK-8-based assessment of cell viability after treatment with different hydrogel components. (c) Live/dead staining of HSFs after treatment with different hydrogel formulations. Green indicates live cells and red indicates dead cells. Scale bar, 50 μm. (d) Live/dead staining of HaCaT cells after treatment with different hydrogel formulations. Scale bar, 50 μm. (e) Hemolysis images and corresponding hemolysis rates of SP, Nap-β, ZnO, and CS-SP-Nap-β@ZnO. Physiological saline and 0.1% Triton X-100 were used as negative and positive controls, respectively. Data are expressed as the mean ± SD (n = 3).

Supplementary Figure 7

Figure S7. Supplementary in vitro cellular functional assays and reactive oxygen species scavenging evaluation. (a, b) Flow-cytometry gating strategy for primary macrophages. (c, d) Cell-free scavenging of O₂•⁻ and H₂O₂ by ZnO, with SOD and CAT as positive controls, respectively. (e, f) Representative HaCaT EdU staining images and quantification of cell proliferation. Scale bar, 100 μm. (g, h) Representative HaCaT Transwell images and quantification of migrated cells. Scale bar, 100 μm. Data are expressed as the mean ± SD (n = 3). ns, not statistically significant; *P < 0.05, **P < 0.01, ***P < 0.001, ****P < 0.0001.

Supplementary Figure 8

Figure S8. Supplementary evaluation of the UVB-induced delayed-healing wound model and preliminary in vivo biosafety. (a) Representative images showing UVB-induced dorsal skin injury. (b) H&E and Masson's staining of normal skin, UVB-irradiated skin, and UVB-irradiated wound tissue. (c–f) Serum TNF-α and IL-6 levels on days 14 and 21 after treatment. (g) Serum SOD activity on day 3 after treatment. (h–j) Serum ALT, UREA, and Zn²⁺ levels on day 21. Data are expressed as the mean ± SD (n = 3 rats per group). ns, not statistically significant; *P < 0.05, **P < 0.01, ***P < 0.001, ****P < 0.0001.

Supplementary Figure 9

Figure S9. H&E staining of major organs following treatment.

Table S1 Predicted protease cleavage profile of substance P

| Enzyme | Number of predicted cleavages | Cleavage-site positions | | Peptide sequence |
| --- | --- | --- | --- | --- |
| Arg-C proteinase | 1 | 1 | RPKPQQFFGLM | |
| CNBr | 1 | 11 | RPKPQQFFGLM | |
| Chymotrypsin-high specificity | 2 | 7 8 | RPKPQQFFGLM | |
| Chymotrypsin-low specificity | 4 | 7 8 10 11 | RPKPQQFFGLM | |
| Clostripain | 1 | 1 | RPKPQQFFGLM | |
| LysC | 1 | 3 | RPKPQQFFGLM | |
| LysN  Pepsin (pH1.3)  Pepsin (pH>2)  Proline-endopeptidase [*]  Proteinase K  Thermolysin | 1  5  5  2  3  4 | 2  6 7 8 9 10  6 7 8 9 10  2 4  7 8 10  6 7 9 10 | RPKPQQFFGLM  RPKPQQFFGLM  RPKPQQFFGLM  RPKPQQFFGLM  RPKPQQFFGLM  RPKPQQFFGLM | |

Table S2 Predicted target sites for the substance P fragment RPKPQQ

| Target | Gene/common name | UniProt ID | ChEMBL ID | Target class | Probability* |
| --- | --- | --- | --- | --- | --- |
| Cyclin-dependent kinase 2/cyclin A complex | CDK2 CCNA1 CCNA2 | P24941 P78396 P20248 | CHEMBL2094128 | Other intracellular protein | 0.932911582 |
| Neurotensin receptor 1 | NTSR1 | P30989 | CHEMBL4123 | Class A G protein-coupled receptor | 0.9268983 |
| HLA class I histocompatibility antigen A-3 | HLA-A | P04439 | CHEMBL2632 | Cell-surface antigen | 0.52099319 |
| Renin | REN | P00797 | CHEMBL286 | Protease | 0.41898222 |
| Integrin alpha-V/beta-3 | ITGAV ITGB3 | P06756 P05106 | CHEMBL1907598 | Membrane-associated receptor | 0.31763241 |
| Integrin alpha-IIb/beta-3 | ITGA2B ITGB3 | P08514 P05106 | CHEMBL2093869 | Membrane-associated receptor | 0.31763241 |
| Inhibitor of apoptosis protein 3 | XIAP | P98170 | CHEMBL4198 | Other intracellular protein | 0.31763241 |
| Thrombin | F2 | P00734 | CHEMBL204 | Protease | 0.31763241 |
| Hepatocyte growth factor activator | HGFAC | Q04756 | CHEMBL3351190 | Unclassified target | 0.21606519 |
| Neuropilin-1 (by homology) | NRP1 | O14786 | CHEMBL5174 | Secreted protein | 0.21606519 |
| Neurotensin receptor 2 | NTSR2 | O95665 | CHEMBL2514 | Class A G protein-coupled receptor | 0.21606519 |

Table S3 Predicted target sites for the substance P fragment RPKPQQF

| Target | Gene/common name | UniProt ID | ChEMBL ID | Target class | Probability* |
| --- | --- | --- | --- | --- | --- |
| Cyclin-dependent kinase 2/cyclin A complex | CDK2 CCNA1 CCNA2 | P24941 P78396 P20248 | CHEMBL2094128 | Other intracellular protein | 0.322951269 |
| Inhibitor of apoptosis protein 3 | XIAP | P98170 | CHEMBL4198 | Other intracellular protein | 0.169888699 |
| Thrombin | F2 | P00734 | CHEMBL204 | Protease | 0.150577867 |
| Neurotensin receptor 1 (by homology) | NTSR1 | P30989 | CHEMBL4123 | Class A G protein-coupled receptor | 0.131513025 |
| Bradykinin B1 receptor | BDKRB1 | P46663 | CHEMBL4308 | Class A G protein-coupled receptor | 0.112222066 |
| HLA class I histocompatibility antigen A-3 | HLA-A | P04439 | CHEMBL2632 | Cell-surface antigen | 0.102431864 |
| Neurokinin 1 receptor (by homology) | TACR1 | P25103 | CHEMBL249 | Class A G protein-coupled receptor | 0.102431864 |
| Neurotensin receptor 2 | NTSR2 | O95665 | CHEMBL2514 | Class A G protein-coupled receptor | 0.083326526 |
| μ-opioid receptor | OPRM1 | P35372 | CHEMBL233 | Class A G protein-coupled receptor | 0.083326526 |
| δ-opioid receptor | OPRD1 | P41143 | CHEMBL236 | Class A G protein-coupled receptor | 0.083326526 |

Table S4 Predicted target sites for the substance P fragment RPKPQQFF

| Target | Gene/common name | UniProt ID | ChEMBL ID | Target class | Probability* |
| --- | --- | --- | --- | --- | --- |
| Cyclin-dependent kinase 2/cyclin A complex | CDK2 CCNA1 CCNA2 | P24941 P78396 P20248 | CHEMBL2094128 | Other intracellular protein | 0.274203829 |
| Thrombin | F2 | P00734 | CHEMBL204 | Protease | 0.150577867 |
| Neurotensin receptor 1 (by homology) | NTSR1 | P30989 | CHEMBL4123 | Class A G protein-coupled receptor | 0.131513025 |
| Bradykinin B1 receptor | BDKRB1 | P46663 | CHEMBL4308 | Class A G protein-coupled receptor | 0.112222066 |
| μ-opioid receptor | OPRM1 | P35372 | CHEMBL233 | Class A G protein-coupled receptor | 0.112222066 |
| δ-opioid receptor | OPRD1 | P41143 | CHEMBL236 | Class A G protein-coupled receptor | 0.112222066 |
| Neurotensin receptor 2 | NTSR2 | O95665 | CHEMBL2514 | Class A G protein-coupled receptor | 0.102431864 |
| κ-opioid receptor | OPRK1 | P41145 | CHEMBL237 | Class A G protein-coupled receptor | 0.102431864 |
| Inhibitor of apoptosis protein 3 | XIAP | P98170 | CHEMBL4198 | Other intracellular protein | 0.102431864 |
| Neuropeptide FF receptor 2 | NPFFR2 | Q9Y5X5 | CHEMBL5952 | Class A G protein-coupled receptor | 0.073884556 |
| Angiotensin II receptor | AGTR2 | P50052 | CHEMBL4607 | Class A G protein-coupled receptor | 0.073884556 |

Table S5 Predicted target sites for the substance P fragment RPKPQQFFG

| Target | Gene/common name | UniProt ID | ChEMBL ID | Target class | Probability* |
| --- | --- | --- | --- | --- | --- |
| Cyclin-dependent kinase 2/cyclin A complex | CDK2 CCNA1 CCNA2 | P24941 P78396 P20248 | CHEMBL2094128 | Other intracellular protein | 0.401277808 |
| Neurotensin receptor 1 | NTSR1 | P30989 | CHEMBL4123 | Class A G protein-coupled receptor | 0.198885519 |
| Thrombin | F2 | P00734 | CHEMBL204 | Protease | 0.131513025 |
| Bradykinin B1 receptor | BDKRB1 | P46663 | CHEMBL4308 | Class A G protein-coupled receptor | 0.112222066 |
| Inhibitor of apoptosis protein 3 | XIAP | P98170 | CHEMBL4198 | Other intracellular protein | 0.112222066 |
| Angiotensin II receptor | AGTR2 | P50052 | CHEMBL4607 | Class A G protein-coupled receptor | 0.092751834 |
| Neurotensin receptor 2 | NTSR2 | O95665 | CHEMBL2514 | Class A G protein-coupled receptor | 0.083326526 |
| μ-opioid receptor | OPRM1 | P35372 | CHEMBL233 | Class A G protein-coupled receptor | 0.083326526 |
| δ-opioid receptor | OPRD1 | P41143 | CHEMBL236 | Class A G protein-coupled receptor | 0.083326526 |
| κ-opioid receptor | OPRK1 | P41145 | CHEMBL237 | Class A G protein-coupled receptor | 0.083326526 |
| Angiotensin-converting enzyme | ACE | P12821 | CHEMBL1808 | Protease | 0.073884556 |
| Melanocortin receptor 4 | MC4R | P32245 | CHEMBL259 | Class A G protein-coupled receptor | 0.073884556 |

Table S6 Quantitative analysis of SP and Nap-β conjugation onto chitosan

| Peptide | Grafting efficiency (%) | Grafted amount (mg⁻¹ chitosan) | Molar degree of substitution (%) |
| --- | --- | --- | --- |
| SP | 61.18 ± 1.88 | 0.612 ± 0.019 | 0.0075 ± 0.0002 |
| Nap-β | 46.46 ± 2.70 | 4.646 ± 0.270 | 0.0821 ± 0.0048 |
